# Is there evidence that walking groups have health benefits? A systematic review and meta-analysis

**DOI:** 10.1136/bjsports-2014-094157

**Published:** 2015-01-19

**Authors:** Sarah Hanson, Andy Jones

**Affiliations:** Norwich Medical School, University of East Anglia, Norwich, Norfolk, UK

**Keywords:** Walking, Physical activity

## Abstract

**Objective:**

To assess the health benefits of outdoor walking groups.

**Design:**

Systematic review and meta-analysis of walking group interventions examining differences in commonly used physiological, psychological and well-being outcomes between baseline and intervention end.

**Data sources:**

Seven electronic databases, clinical trial registers, grey literature and reference lists in English language up to November 2013.

**Eligibility criteria:**

Adults, group walking outdoors with outcomes directly attributable to the walking intervention.

**Results:**

Forty-two studies were identified involving 1843 participants. There is evidence that walking groups have wide-ranging health benefits. Meta-analysis showed statistically significant reductions in mean difference for systolic blood pressure −3.72 mm Hg (−5.28 to −2.17) and diastolic blood pressure −3.14 mm Hg (−4.15 to −2.13); resting heart rate −2.88 bpm (−4.13 to −1.64); body fat −1.31% (−2.10 to −0.52), body mass index −0.71 kg/m^2^ (−1.19 to −0.23), total cholesterol −0.11 mmol/L (−0.22 to −0.01) and statistically significant mean increases in VO_2__max_ of 2.66 mL/kg/min (1.67–3.65), the SF-36 (physical functioning) score 6.02 (0.51 to 11.53) and a 6 min walk time of 79.6 m (53.37–105.84). A standardised mean difference showed a reduction in depression scores with an effect size of −0.67 (−0.97 to −0.38). The evidence was less clear for other outcomes such as waist circumference fasting glucose, SF-36 (mental health) and serum lipids such as high-density lipids. There were no notable adverse side effects reported in any of the studies.

**Conclusions:**

Walking groups are effective and safe with good adherence and wide-ranging health benefits. They could be a promising intervention as an adjunct to other healthcare or as a proactive health-promoting activity.

## Introduction

Regular physical activity positively impacts health potentially offering similar effects to some drug interventions in terms of mortality benefits. Indeed, it has been suggested as an alternative or adjunct to conventional drug therapy.^1^ Walking at a pace of 3–5 m/h (5–8 km/h) expends sufficient energy to be classified as moderate intensity^2^ and is an easy and accessible way of meeting physical activity recommendations.^3^ Systematic reviews and meta-analyses have shown walking to have various health benefits including positive effects on fitness, fatness and resting blood pressure,^4^ blood pressure control,^5^ weight loss,^6^ depression^7^ and cardiovascular disease risk prevention.^8^

Despite evidence and government campaigns such as Change4life^9^ to promote physically active lifestyles, few are active enough to be of benefit to general health. In England, for example, 29% of adults do less than 30 min of moderate physical activity per week^10^ and about 8% do not even walk continuously for 5 min over 4 weeks.^11^ The impact of interventions in primary care to reduce inactivity appears limited; simple advice to be more active has only moderate yet short-term effects and an effective way of increasing physical activity and improving associated health indicators while also making the most efficient use of doctors’ resources has yet to be determined.^12–14^

One way to promote and sustain walking behaviours at the population level may be through the provision of outdoor walking groups.^15^ Walking groups are typically short walks of under an hour in the natural environment, run by trained lay people. An example of such is ‘Walking for Health’, a scheme originally set up by an Oxford General Practitioner in 2000. It is England's largest network of lay-led health group walks with 70 000 regular walkers, 10 000 volunteer walk leaders and approximately 3000 short walks offered every week.^16^ Group walking is a potentially attractive physical activity intervention that has particular potential to engage those who are interested in the outdoors, whether for leisure or as a health intervention and has been found to be cost-effective in increasing physical activity.^17^ Additionally, the dynamics and social cohesion of walking groups may have supportive effects that encourage and sustain adherence and positive attitudes towards physical activity,^18^ companionship and a shared experience of wellness.^19^ A systematic review in 2007 by Ogilvie *et al*^20^ concluded that people could be encouraged to walk more if interventions were tailored to their needs and targeted at the most sedentary or at those most motivated to change and that group-based approaches, such as the social support of walking groups, are one method of delivering this. In a recent review, walking groups were found to be efficacious at increasing physical activity, particularly when targeted at older adults.^21^ However, it remains that the benefits to health from increasing physical activity are greater than increasing fitness levels, yet no review to date has attempted to quantify the wider health benefits of walking groups. Hence, this review has been undertaken to understand whether there is evidence that outdoor walking groups show wider health benefits as an intervention and therefore could be recommended by clinicians.

## Methods

This systematic review followed Cochrane systematic review guidelines,^22^ requirements of the NHS National Institute of Health Research Centre for Reviews and Dissemination^23^ and the PRISMA statement for reporting studies that evaluate healthcare interventions.^24^ ^25^ Methods of the analysis and inclusion criteria were specified in advance and documented in a protocol registered as CRD42013006397^23^ available at http://www.crd.york.ac.uk/prospero/

### Data sources

We searched using electronic databases; clinical trials registers; by scanning reference lists of articles and from grey literature. For the electronic databases, the search with specific search terms was applied in to AMED, EMBASE, MEDLINE (R) in process and other non-indexed citations and PsycINFO (sourced through OVID); SportDiscus and CINAHL (sourced through EBSCO) and SCOPUS with no date restriction. Databases were selected to best represent source material in health, allied health, physical activity and human science. Clinical trials registers were searched through the UK clinical trials research network study portfolio; clinicaltrials.gov and controlledtrials.com. Grey literature included reports from Natural England, Walking for Health and the National Institute for Health and Care Excellence. Additionally, reference lists from included studies and systematic reviews on exercise and walking were hand searched. The search was completed in November 2013.

Inclusion criteria were studies of outdoor walking groups involving adults with measured physiological, psychological or well-being outcomes. The search was restricted to papers published in English. The inclusion criteria are further detailed in table 1.

**Table 1:** Inclusion and exclusion criteria

Inclusion	Exclusion
Adults from the age of 19	Youths and children up to and including 18
Interventions where people walk as part of a defined walking group intervention	Studies that do not involve a walking group intervention, eg, they walk with a physiotherapist
Where the walking is group based, or where the walking is predominantly group based but participants may also walk on their own to supplement this	Participants walking only rarely in groups, or walking on their own, such as home-based or pedometer-based programmes with no group walking
Walking outdoors or walking predominantly outdoors but occasionally indoors (eg, inside tracks or shopping malls for weather reasons)	Walking indoors or predominantly indoors
Studies that compare group walking with group Nordic walking where group walking can be isolated as an intervention and the outcome directly related to group walking	Studies examining Nordic walking only
Studies with physiological, psychological or well-being outcomes such as blood profiles (eg, lipids, HbA1c), cardiovascular measures (eg, BP), psychological (eg, Beck depression inventory), well-being (eg, EQ5D)	Studies where the outcomes are solely physical activity such as step outcomes or logs of physical activity
Studies where the outcome can directly be related to the walking group intervention	Studies with a mixed intervention (eg, walking with calcium supplements or walking combined with a health education intervention) where the outcome cannot be isolated and directly attributed to group walking
Papers and documents written in English	Papers and documents not written in English

Search terms were developed with reference to the previous systematic reviews on walking^20^ ^21^ and key words from relevant studies. They were piloted to ensure that known studies were identified. The search syntax for the electronic databases is detailed in online supplementary information appendix (i). For clinical trials registers, the only search term was ‘walking’ within the title.

### Study selection

All studies where the outcome could be directly attributable to the group walking were included. This included studies where walking was the control group. All studies were reviewed by the first reviewer and duplicates or the clearly irrelevant, for example, walk-in centres, using wii-fit, or studies using children or animals that had not been screened out by the database filters were excluded. A particular issue with the assessment of the studies was that the phrase ‘walking group’ often related to a walking arm of a study, or a group within a trial that could walk, and not a ‘walking group’ per se. Additionally, there was commonly little information within the abstract about the setting of the intervention, for example, treadmill or indoor circuit-based interventions or home-based solo interventions with physical activity diaries and pedometers. Therefore, most studies were retrieved as full texts and scanned for intervention information to ensure that none were excluded incorrectly. Owing to the generally poor description of the intervention, 40 authors were contacted to confirm whether the study was an outdoor intervention and that they walked as a group. To further ensure that studies had been correctly excluded, 15% of the excluded studies were selected by random number generation and screened by the second author (AJ). All papers were found to have been excluded correctly and therefore no further excluded studies were reviewed.

### Data extraction

A data extraction sheet was developed by both authors to summarise the study, the population, walking group characteristics, the intervention (volume and intensity), adherence and outcomes. This was piloted on five manuscripts and refined accordingly. Data were extracted by the first reviewer into a coding frame using Microsoft Excel, synthesised and tabulated.

### Risk of bias in individual studies and across studies within meta-analyses

As not all studies were randomised controlled trials, a tool used by Ogilvie *et al*^20^ was adapted to assess risk of bias and internal validity^26^ with nine items on a binary scale. These were:

1. Randomisation: Was there sufficient description of a randomisation process or statistical test to show that comparability between the two groups has been adjusted for (no explanation scores scored zero)?
2. Exposure: Did the authors show that there was no evidence of a concurrent intervention which could have influenced the results (no explanation scores zero)?
3. Representativeness: Were the study samples shown to be representative of the study population?
4. Comparability: Were baseline characteristics of the intervention comparable with the control or were potential confounders at baseline appropriately adjusted for in analysis?
5. Attrition: Were numbers of participants at follow-up identifiable as at least 80% of the baseline?
6. Follow-up tools: Were valid and reliable tools used to assess participant outcomes?
7. Follow-up time scale: Was the time to follow-up assessment of a period no less than 1 month?
8. Precision of the results: Were CIs or p values given?
9. Was there evidence presented that the study was sufficiently powered at follow-up assessment (no evidence or underpowered scores zero)?

Publication bias across studies within the meta-analysis was tested with funnel plots using SE as the measure of study size on the vertical axis^27^ and mean difference on the horizontal.

### Synthesis of results and statistical analysis

Data for the final studies were synthesised with results for each study recorded as change from baseline to the end of the intervention (↑↓) with p values where available. Non-significant or imprecise p values, such as p>0.05, were used only when this was the only available information. No assumptions were made about walking outside the group provision. To establish the mean difference between baseline and the end of intervention for meta-analysis, baseline data with SD and sample size, and end of intervention data with SD or SE and sample size were utilised. All data were continuous and a difference in means was used except for one analysis; for depression a standardised mean difference was used to account for the different outcome measurements used in the five studies. There was no need for data to be transformed as a reduction in value indicated an improvement in health in all four outcome measures within this analysis. A fixed effects model was used for all analyses representing a more conservative measure than a random effects model.^22^ Where data were given for different subgroups, each was input separately and combined in meta-analyses using the RevMan software package.^28^ All results are presented with 95% CIs. The I² statistic was used to test for heterogeneity. I^2^ values of 30–60% and 50–90% were taken to represent moderate and substantial heterogeneity, respectively (ref. 22, Ch 9.5.2).

## Results

The initial database search yielded 5145 citations. In addition, the other supplementary sources produced a further 60 studies. Of these 5205 studies, 4627 were removed as duplicates or as clearly irrelevant after reviewing titles. The abstracts of 578 articles were screened and any that did not provide enough information were retrieved for full-text evaluation. A total of 150 papers were read as full texts to be assessed for eligibility. The remaining 46 articles were put forward for second review and independently assessed by the second reviewer (AJ). From this, 10 papers were discussed between the two reviewers. Three studies were excluded due to a lack of information despite repeated attempts to contact authors as the reviewers lacked confidence that the intervention was group based and outdoors. One was excluded on further discussion due to the walking being primarily self-directed. In total, 42 studies met the inclusion criteria and were eligible to be included in the synthesis. Walking groups were used as a control in seven of the studies. The review flow chart is detailed in online supplementary figure S1. The characteristics and synthesised results from all 42 studies are detailed in online supplementary table S1.

All 42 studies were assessed for risk of bias (table 2). No study was excluded due to a low-quality score. Assessments of quality were made by the first reviewer and 20% of the studies were chosen by random number generation and checked by the second reviewer. An inter-rater reliability analysis using the κ statistic was performed to determine consistency among raters and found to be κ 0.66 (p<0.001) representing substantial agreement.

**Table 2:** Risk of bias for included studies

		Risk of bias items									Total score
Author	Study type	1	2	3	4	5	6	7	8	9	
Armstrong	RCT	1	0	1	1	1	1	1	0	1	7
Bjersing	RCT	1	0	1	1	1	1	1	1	0	7
Brandon	RCT	0	1	1	1	1	1	1	1	1	8
Brosseau	RCT	1	0	1	1	0	1	1	1	0	6
Cox	RCT	1	1	1	1	1	1	1	1	1	9
Duncan	RCT	0	1	1	1	1	1	1	1	0	7
Fisher	RCT	1	0	1	1	0	1	1	1	0	6
Gusi	RCT	1	0	1	1	1	1	1	1	1	8
Hamdorf	RCT	1	1	1	1	0	1	1	1	0	7
Hinkleman	RCT	0	1	1	1	0	1	1	1	0	6
Isaacs	RCT	1	1	1	1	1	1	1	1	1	9
Kamijo	RCT	0	1	1	1	1	1	1	1	0	7
Kayo	RCT	1	0	1	1	0	1	1	1	1	7
Legrand	RCT	0	0	1	1	1	1	1	1	0	6
Mannerkorpi	RCT	1	1	1	1	0	1	1	1	1	8
Moore-Harrison	RCT	0	0	1	1	1	1	1	1	0	6
Morrison	RCT	0	0	1	1	1	1	1	1	0	6
Negri	RCT	1	0	1	1	0	1	1	1	0	6
Palmer	RCT	0	1	1	1	1	1	1	0	0	6
Reuter	RCT	1	1	1	1	1	1	1	1	1	9
Rooks	RCT	1	1	1	1	1	1	1	1	0	8
van Uffelen	RCT	1	0	1	1	1	1	1	1	0	7
Callahan	Pre–post	0	0	1	1	1	1	1	1	0	6
Dallocchio	Pre–post	0	0	1	1	1	1	1	1	0	6
Fantin	Pre–post	0	0	1	1	1	1	1	1	1	7
Figard-Fabre	Pre–post	0	1	1	1	1	1	1	1	0	7
Gelecek	Pre–post	0	1	1	1	1	1	1	0	0	6
Holmberg	Pre–post	0	0	1	1	0	1	1	1	0	5
Moss	Pre–post	0	0	1	1	1	1	1	0	0	5
O'Halloran	Pre–post	0	0	1	1	1	1	1	0	0	5
O'Hara	Pre–post	0	0	1	0	1	1	1	0	0	4
Cavanaugh	CT	0	1	1	1	1	1	1	1	0	7
Fritz	CT	0	0	1	0	1	1	1	1	0	5
Park	CT	0	1	1	1	0	1	1	1	0	6
Roberts	CT	0	1	1	1	1	1	1	0	0	6
Silverthorn	CT	0	1	1	1	1	1	1	0	0	6
Song	CT	1	0	1	1	1	1	1	1	1	8
Takahashi	CT	0	1	0	1	1	1	1	1	0	6
Thomas	CT	0	0	1	1	1	0	1	0	0	4
Cyarto	Quasi-experimental	0	1	1	1	1	1	1	1	0	7
McDevitt	Quasi-experimental	0	0	1	1	1	1	1	1	0	6
Ng	Cohort study	0	0	1	0	1	1	1	0	0	4

(1) Randomisation, (2) exposure, (3) representativeness, (4) comparability, (5) attrition, (6) follow-up tools, (7) follow-up time scale, (8) precision of the results, (9) statistical power.

Grey scale indicates studies included in meta-analysis.

CT, controlled trial; RCT, randomised controlled trial.

### Study characteristics

Although there was no date restriction on the search, 74% of the articles were studies in the past 10 years suggesting the recent interest in walking groups, with no papers prior to 1988 meeting the inclusion criteria. Studies were located in 14 different countries but predominantly in the USA (n=15). A total of 1843 participants walked in outdoor walking groups with at least 1488 h of provision (3 studies did not give enough information from which to calculate dosage) and a total of 74 023 h of participant walking time. Walking groups were used with participants with a broad range of health conditions: arthritis,^29^ ^30^ dementia and cognitive impairment,^31–33^ diabetes,^34–36^ fibromyalgia,^37–39^ obesity and overweight,^40–44^ mental health issues^45–49^ and Parkinson's disease^50^ with 64 different tools used to test outcomes.

In terms of participants, 76% were women while 43% of the studies were for women only; there were no studies for men only. The grand mean age was 58 years with 15 studies specifically aimed at older participants. There was subanalysis in four studies: ethnicity,^40^ intensity^47^ ^51^ and gender.^43^ Two studies were of people with learning disabilities living in care facilities: one obese adults with Prader-Willi syndrome,^52^ and the second the coronary heart disease risk of adults with learning disabilities.^43^ Eleven studies described the ethnicity of the participants and 13 studies provided some socioeconomic information. Brandon and Elliott-Lloyd^40^ compared the response between African-American women, and the O’Hara *et al*^44^ study was specifically for African-American women. Otherwise there was no evaluation of effect for different ethnicities.

Interventions were varied, in volume and intensity, ranging from 168 to 8580 min of walking over a period of 3 weeks to 1 year, with intensity ranging from self-selected and low to brisk walking and high-intensity intervals. Moore-Harrison *et al*^53^ specifically targeted those of low socioeconomic profile, and Isaacs *et al*^54^ provide subanalysis of uptake of walking group intervention by socioeconomic status. Where supervision was described, it was by professionals, such as physiotherapists, possibly as the interventions were part of clinical trials. Where described, provision was in rural locations in 6 of the studies, and urban for 15. Where additional information from authors has been obtained, this has been added to the results table 2 (see online supplementary information). Adherence and adverse effects are described in 76% of the papers. Mean adherence (where stated) was 75%. One study notes that adherence was lower for those without access to private transport.^54^ For adverse effects, one study described one fall with a brief absence from the walking programme,^55^ one a calf injury^46^ and one, a study with participants with Parkinson's disease, describes one participant experiencing exercise-induced hypotension after intense uphill walking in hot weather and four falls on roots and wet ground.^50^ Otherwise, either authors state that there were no injuries, or there is no reference to adverse effects. This is against a back drop of over 74 000 participant hours. Attrition was less clearly described but in one study there was a participant withdrawal as overweight and self-conscious;^45^ one author states that travel to the walking club may have affected attrition,^29^ and one describes the different attrition rates between African-American and white walkers.^40^

### Meta-analysis

Common outcome measures enabled meta-analysis of 17 frequently used outcome measures, summarised in table 3 and presented in full in online supplementary information appendix (iii). Statistically significant improvements from baseline to end of intervention were identified for participants in the intervention groups for systolic and diastolic blood pressure, resting heart rate, body fat, body mass index (BMI), total cholesterol, VO_2__max_, quality of life for physical functioning, 6 min walk time and depression. For depression, a standardised mean difference of −0.67 (−0.97 to −0.38) represents a statistically significant moderate effect.^22^ For other outcomes, the effects were not statistically significant.

**Table 3:** Summary meta-analysis results table: difference between baseline and end of intervention

Outcome measure	n	Effect	95% CIs	Heterogeneity	Test for overall effect
Systolic BP (mm Hg)	440	−3.72	(−5.28 to −2.17)	χ^2^=12.02, df=12 (p=0.44); I²=0%	z=4.70 (p<0.001)
Diastolic BP (mm Hg)	440	−3.14	(−4.15 to −2.13)	χ^2^=23.16, df=12 (p=0.03); I²=48%	z=6.09 (p<0.001)
Resting HR (bpm)	252	−2.88	(−4.13 to −1.64)	χ^2^=2.96, df=7 (p=0.89); I²=0%	z=4.53 (p<0.001)
Body fat (%)	328	−1.31	(−2.10 to −0.52)	χ^2^=4.00, df=6 (p=0.68); I²=0%	z=3.25 (p=0.001)
Body mass index (kg/m^2^)	451	−0.71	(−1.19 to −0.23)	χ^2^=5.52, df=11 (p=0.90); I²=0%	z=2.92 (p=0.003)
Total cholesterol (mmol/L)	271	−0.11	(−0.22 to −0.01)	χ^2^=12.58, df=9 (p=0.18); I²=28%	z=2.13 (p=0.03)
VO_2max_ (mL/kg/min)	166	2.66	(1.67 to 3.65)	χ^2^=9.67, df=6 (p=0.14); I²=38%	z=5.28 (p<0.001)
SF-36 score (physical functioning) (points)	68	6.02	(0.51 to 11.53)	χ^2^=0.26, df=1 (p=0.61); I²=0%	z=2.14 (p=0.03)
6 min walk time (m)	65	79.6	(53.37 to 105.84)	χ^2^=0.71, df=1 (p=0.40); I²=0%,	z=5.95 (p≤0.001)
Depression score*(effect size)	101	−0.67	(−0.97 to −0.38)	χ^2^=24.14, df=4 (P≤0.001); I²=83%	z=4.44 (p≤0.001)
Waist circumference (cm)	35	−3.55	(−8.08 to 0.98)	χ^2^=0.52, df=1 (p=0.47); I²=0%	z=1.54 (p=0.12)
HbA1c (%)	66	−0.11	(−0.25 to 0.03)	χ^2^=1.17, df=3 (p=0.76); I²=0%	z=1.53 (p=0.13)
Fasting glucose (mmol/L)	85	−0.09	(−0.28 to 0.11)	χ^2^=3.33, df=4 (p=0.50); I²=0%	z=0.87 (p=0.38)
Low-density lipids (mmol/L)	268	−0.05	(−0.16 to 0.06)	χ^2^=8.83, df=9 (p=0.45); I²=0%,	z=0.93 (p=0.35)
High-density lipids (mmol/L)	251	0.01	(−0.04 to 0.07)	χ^2^=8.04, df=8 (p=0.43); I²=0%	z=0.45 (p=0.65)
Triglycerides (mmol/L)	271	−0.05	(−0.12 to 0.03)	χ^2^=13.39, df=9 (p=0.15); I²=33%	z=1.25 (p=0.21)
SF-36 score (mental health index) (points)	68	2.70	(−2.09 to 7.48)	χ^2^=0.18, df=1 (p=0.67); I²=0%	z=1.10 (p=0.27)

*All analyses fixed effects model and mean difference except depression score (effect is standardised mean difference).

BP, blood pressure; HbA1c, glycated haemoglobin; HR, heart rate.

There was zero heterogeneity in 12 of the analyses with 4 having an I^2^ between 28% and 48%. The depression score had an I^2^ of 83% suggesting a high level of heterogeneity between the studies. Using funnel plots, all studies were visually symmetrical with a narrow spread at the top of the funnel indicating precision with results close to the pooled estimate and without bias towards smaller studies (see online supplementary appendix ii).

In order to test if the impact of the group walking was greater in those with clearly defined morbidity, a sub analysis was completed for the conditions of overweight or obese (BMI≥25), Type II diabetes (as defined by authors) and depression (as defined by authors). For depression and BMI this strengthened the results. By only including those defined as depressed^17^ ^45^ ^47^ the effect size became large −0.76 (−1.12 to −0.41). By only including those with a BMI ≥25^17^ ^34^ ^35^ ^40^ ^41^ ^43^ ^54^ ^56^ ^57^ the mean difference increased to −0.75 (−1.26 to −0.24). For glycated haemoglobin (HbA1c) and fasting glucose, only including those with type II diabetes^34^ ^35^ the mean differences remained statistically not significant −0.16 (−0.40 to 0.08) and −0.57 (−1.58 to 0.43) respectively.

## Discussion

### Principal findings

This systematic review and meta-analysis provides evidence that outdoor walking groups have health benefits over and above making people more physically active. Statistically significant improvements were found in a range of widely used measures of health; systolic and diastolic blood pressure, resting heart rate, body fat, BMI, total cholesterol, VO_2__max_, depression, 6-min walk time, and quality of life for physical functioning. This is despite the fact that the majority of the interventions (75%) were below international moderate activity guidelines which may account for some of the effect sizes being small. Walking groups appear an acceptable intervention to participants with high levels of adherence and a low risk of serious adverse effects.

### Strengths and limitations

The strength of this review is that it has comprehensively sought out walking group studies. It has extensively analysed 42 different studies with 1843 participants involved in over 74 000 participant hours of group walking. It has also extracted information for 17 meta-analyses to provide evidence of health benefits and within these was generally zero or low heterogeneity. Limitations of the study are that only manuscripts published in English were sought. Additionally, the populations in the included studies are very different with many small studies. The lack of information on walking dose in many of the studies mean it was not possible to undertake an analysis of dose–responses.

### Results in context of other published reviews

Kassavou *et al*^21^ found that walking groups increase physical activity. The results from this study extend these findings by providing evidence of the wide-ranging health benefits of group walking.

Clinicians and therapists may however be asked whether walking in groups has similar health benefits than walking per se or the use of a pedometer, a widely used method of increasing walking. To explore this, the results of the meta-analysis within this study were compared first with meta-analyses of walking and then with pedometers.

In terms of depression, Robertson *et al*^7^ in their meta-analysis of walking using a fixed effects model, found a standardised mean effect size of −0.86 (−1.12 to −0.61), comparable to the effect size of −0.67 (−0.97 to −0.38) in this review of group walking. In terms of cardiovascular health, a systematic review by Murphy *et al*^4^ of walking using a random effects model found statistically significant reductions in body fat, BMI and diastolic blood pressure and increases in VO_2max_. The effects were however of a smaller magnitude than those found in this study; a reduction of diastolic blood pressure of 1.54 mm Hg from walking compared with 3.14 mm Hg in group walking; a reduction in BMI of 0.2 kg/m² compared with 0.7 kg/m^2^; and a reduction of body fat of 0.63% from walking compared with a reduction of 1.31% in group walking. In addition, Murphy *et al* did not find a statistically significant reduction in systolic blood pressure (−1.06 mm Hg, p=0.316) from walking in contrast to the significant reduction in systolic blood pressure (−3.72 mm Hg, p<0.001) found from group walking in this review. Murphy *et al*^4^ stated a relative reduction of 0.8% in systolic and 2% in diastolic blood pressure. This is comparable to a previous meta-analysis of walking and resting blood pressure^58^ which found a 2% reduction in systolic and diastolic from walking. In comparison, this review of group walking found reductions of 3% in systolic 5% in diastolic blood pressure, representing a greater reduction than those from walking alone. The importance of this difference becomes significant when viewed against findings that a 2 mm Hg in diastolic blood pressure can reduce coronary heart disease risk by 6% and stroke and trans-ischaemic attacks by 15%.^59^ Further evidence of the importance of this reduction comes from a meta-analysis of prospective studies which suggested that a persistent reduction in average blood pressure by widely practicable methods could avoid large absolute numbers of premature deaths and disabling strokes and a reduction of only 2 mm Hg in systolic blood pressure could reduce stroke mortality by 10% and mortality from vascular causes in a middle-aged population by 7%.^60^ Outdoor walking groups could be an example of such a practicable method. The second part of this further analysis compared the results from this systematic review of group walking to a systematic review and meta-analysis of pedometers to increase physical activity and improve health outcomes.^61^ Again walking groups were found to have comparable and greater results to those from pedometers in reductions in BMI, systolic and diastolic blood pressure and total cholesterol. This was particularly significant for diastolic blood pressure with the use of pedometers showing a reduction of −0.3 mm Hg (−0.02 to −0.46) compared with walking groups −3.14 mm Hg (−4.15 to −2.13). It should be noted that the two comparator systematic reviews included outdoor group walking as well as other methods (indoors and solo) in their meta-analysis; within the systematic review of pedometers some of the participants may have walked within a workplace group and additionally people who walk in groups invariably walk by themselves too. Therefore, this further analysis is not a straightforward comparison of non-group versus group methods but this comparison has provided some evidence that group walking may have benefits to health at least equal to walking with pedometers and walking per se.

### Conclusions and meaning of the study for clinicians

This systematic review with meta-analysis has found that outdoor walking groups have wide-ranging health benefits. With low levels of attrition, high levels of adherence and virtually no adverse effects this study suggests that walking groups could be a practicable intervention, acceptable to patients as a line of treatment with a potential for both physiological and psychological health benefits. It may provide clinicians with evidence of a further effective option to recommend to those patients who would benefit from increasing moderate physical activity.

### Unanswered questions and further research

One study evaluated the results based on three different walk speeds.^51^ Otherwise, there were insufficient studies meeting moderate activity guidelines from which to conduct a subanalysis and suggest any tentative conclusions about effectiveness of walking groups and time or intensity. It may be that effect sizes could be improved by increasing volume and intensity and this important question remains unanswered. A lack of socioeconomic information prevented analysis of the distribution and effects between different social groups confirming concerns raised by Ogilvie *et al*^20^ that such targeted interventions may be preferentially utilised by better-off groups^62^ and may thereby increase health inequalities.^63^ The issue of equity could be addressed in future research. Additionally, the majority of the studies in this analysis were with people with diagnosed health conditions or cardiovascular disease risk factors; therefore, the potential benefit of walking groups in maintaining good health in healthy populations is not known. Nevertheless, this review has shown that there are wide-ranging health benefits from outdoor walking groups and these appear not to be counterbalanced by an increase in injuries or other adverse side effects.

**What are the new findings?**

- Outdoor walking groups have wide-ranging health benefits including reducing blood pressure, body fat, total cholesterol and risk of depression.
- Outdoor walking groups appear to be an acceptable intervention to participants, with high levels of adherence and virtually no adverse effects.

**How might it impact on clinical practice in the near future?**

Provides clinicians with evidence of a further effective option to recommend to those patients who would benefit from increasing moderate physical activity.

## Supplementary Material

Web appendix

Web figure

Web protocol

Web table
