# Supplementary material for: Is there evidence that walking groups have health benefits? A systematic review and meta-analysis
Source: Br J Sports Med. 2015 Jan 19;49(11):710–5. doi: 10.1136/bjsports-2014-094157 (PMC4453623; doi:10.1136/bjsports-2014-094157)

SUPPLEMENTARY INFORMATION

Appendix (i) Search syntax for electronic databases

1. "walk* program*".af.
2. "walk* intervention".af.
3. "health walk*".af.
4. "nordic walk*".af.
5. "walk* group*".af.
6. "walk* club*".af.
7. "lay led walk*".af.
8. "community based walk*".af.
9. "community walk*".af.
10. "walk* scheme*".af.
11. "walk* for health".af.
12. "group physical activity". af.
13. 1 or 2 or 3 or 4 or 5 or 6 or 7 or 8 or 9 or 10 or 11 or 12
14. remove duplicates from 13
15. limit 14 to "all adult (19 plus years)"
16. limit 15 to English language

af represents all fields
* or $ sign used as truncation wildcard as appropriate to each database

Appendix (ii) Example funnel plot (systolic blood pressure)

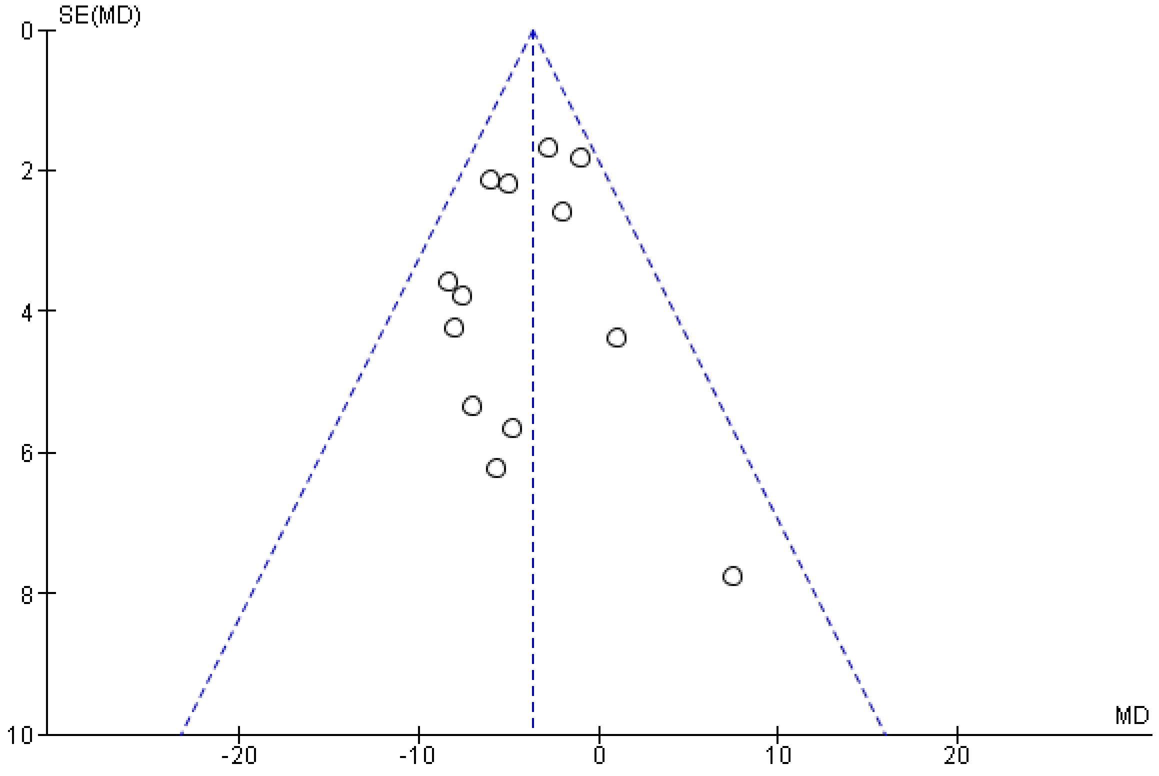

Appendix (iii) Results from Meta-analysis

Systolic blood pressure

Study or Subgroup	WG end of intervention Mean [mmHg]	SD [mmHg]	Total	WG Baseline Mean [mmHg]	SD [mmHg]	Total	Weight	Mean Difference IV, Fixed, 95% CI [mmHg]
Brandon 2006	102.4571	13.733	28	110.7571	13.1328	28	4.9%	-8.30 [-15.34, -1.26]
Duncan 1991	106.3953	9.7117	43	107.3721	6.9813	43	18.9%	-0.98 [-4.55, 2.60]
Fantin 2012	140.27	15.41	21	145.95	23.93	21	1.6%	-5.68 [-17.85, 6.49]
Figard-Fabre 2010	129	12	11	136	13	11	2.2%	-7.00 [-17.45, 3.45]
Fritz 2006	138.6	9.6693	17	146.2	12.2	17	4.4%	-7.60 [-15.00, -0.20]
Gelecek 2006	105	8.5	29	110	8.3	29	12.9%	-5.00 [-9.32, -0.68]
Hamdorf 1999	152.1	25.4558	18	144.6	20.7889	18	1.0%	7.50 [-7.68, 22.68]
Isaacs 2007	133.3	11.7683	103	136.1	21.5149	311	22.2%	-2.80 [-6.10, 0.50]
Morrison 2009	120	8	19	122	8	19	9.3%	-2.00 [-7.09, 3.09]
Moss 2009	111.47	14.4743	100	117.53	15.5923	100	13.9%	-6.06 [-10.23, -1.89]
Negri 2010	134	13.9	21	133	14.5	21	3.3%	1.00 [-7.59, 9.59]
Palmer 1995	112.3	17.7	16	117.1	14	16	2.0%	-4.80 [-15.86, 6.26]
Takahashi 2013	123	11.225	14	131	11.225	14	3.5%	-8.00 [-16.32, 0.32]
**Total (95% CI)**			**440**			**648**	**100.0%**	**-3.72 [-5.28, -2.17]**

Heterogeneity: Chi² = 12.02, df = 12 (P = 0.44); I² = 0%
Test for overall effect: Z = 4.70 (P < 0.00001)

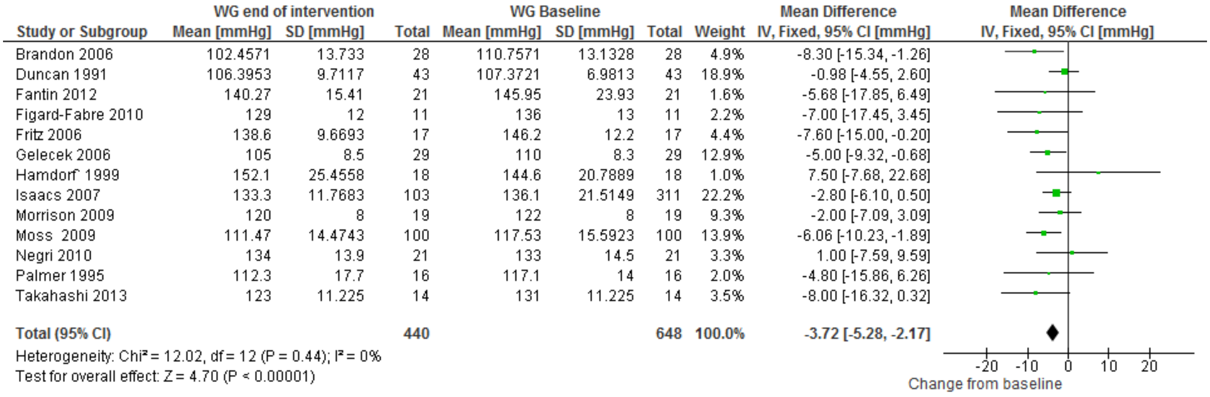

Diastolic blood pressure

Study or Subgroup	WG end of intervention Mean [mmHg]	SD [mmHg]	Total	WG Baseline Mean [mmHg]	SD [mmHg]	Total	Weight	Mean Difference IV, Fixed, 95% CI [mmHg]
Brandon 2006	65.2143	9.5435	28	68.4929	9.4851	28	4.1%	-3.28 [-8.26, 1.71]
Duncan 1991	72.093	6.6505	43	72.3721	8.1373	43	10.4%	-0.28 [-3.42, 2.86]
Fantin 2012	79.18	7.12	21	82.43	10.12	21	3.7%	-3.25 [-8.54, 2.04]
Figard-Fabre 2010	73	7	11	85	7	11	3.0%	-12.00 [-17.85, -6.15]
Fritz 2006	80.9	3.3419	17	85.2	5.6	17	10.7%	-4.30 [-7.40, -1.20]
Gelecek 2006	72	8.7	29	74	6.4	29	6.6%	-2.00 [-5.93, 1.93]
Hamdorf 1999	76.1	8.4853	18	78.6	9.3338	18	3.0%	-2.50 [-8.33, 3.33]
Isaacs 2007	82.1	7.1633	103	84.3	10.0521	311	32.4%	-2.20 [-3.98, -0.42]
Morrison 2009	78	6	19	79	7	19	6.0%	-1.00 [-5.15, 3.15]
Moss 2009	70.94	12.0829	100	76.53	12.9439	100	8.5%	-5.59 [-9.06, -2.12]
Negri 2010	78	7.1	21	80	6.2	21	6.3%	-2.00 [-6.03, 2.03]
Palmer 1995	73.2	9.5	16	80.9	10.6	16	2.1%	-7.70 [-14.67, -0.73]
Takahashi 2013	70	7.4833	14	79	7.4833	14	3.3%	-9.00 [-14.54, -3.46]
**Total (95% CI)**			**440**			**648**	**100.0%**	**-3.14 [-4.15, -2.13]**

Heterogeneity: Chi² = 23.16, df = 12 (P = 0.03); I² = 48%
Test for overall effect: Z = 6.09 (P < 0.00001)

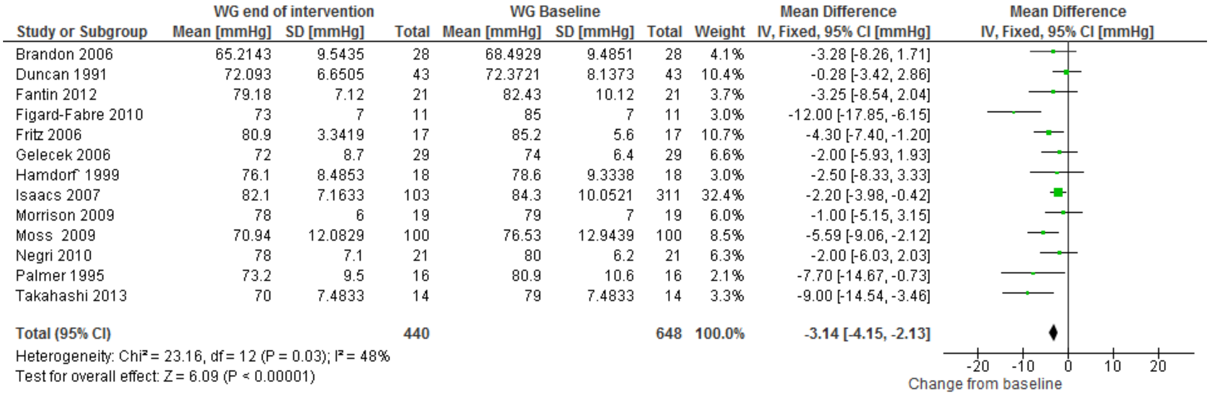

Resting heart rate

Study or Subgroup	WG end of intervention Mean [BPM]	SD [BPM]	Total	WG Baseline Mean [BPM]	SD [BPM]	Total	Weight	Mean Difference IV, Fixed, 95% CI [BPM]
Cox 2006	65.1867	7.54	49	68.7867	7.5433	60	19.2%	-3.60 [-6.45, -0.75]
Fantin 2012	76.5	5.93	21	78.82	7.3	21	9.6%	-2.32 [-6.34, 1.70]
Figard-Fabre 2010	81	16	11	80	12	11	1.1%	1.00 [-10.82, 12.82]
Gelecek 2006	73.8	6.2	29	78.6	6.8	29	13.9%	-4.80 [-8.15, -1.45]
Hamdorf 1999	71.8	8.9095	18	74.4	8.9095	18	4.6%	-2.60 [-8.42, 3.22]
Isaacs 2007	63	4.3359	102	65.2	10.5811	160	45.9%	-2.20 [-4.04, -0.36]
Palmer 1995	72.2	9	16	74	10.8	16	3.3%	-1.80 [-8.69, 5.09]
Silverthorn 1993	56	7.1	6	61.33	7.45	6	2.3%	-5.33 [-13.56, 2.90]
**Total (95% CI)**			**252**			**321**	**100.0%**	**-2.88 [-4.13, -1.64]**

Heterogeneity: Chi² = 2.96, df = 7 (P = 0.89); I² = 0%
Test for overall effect: Z = 4.53 (P < 0.00001)

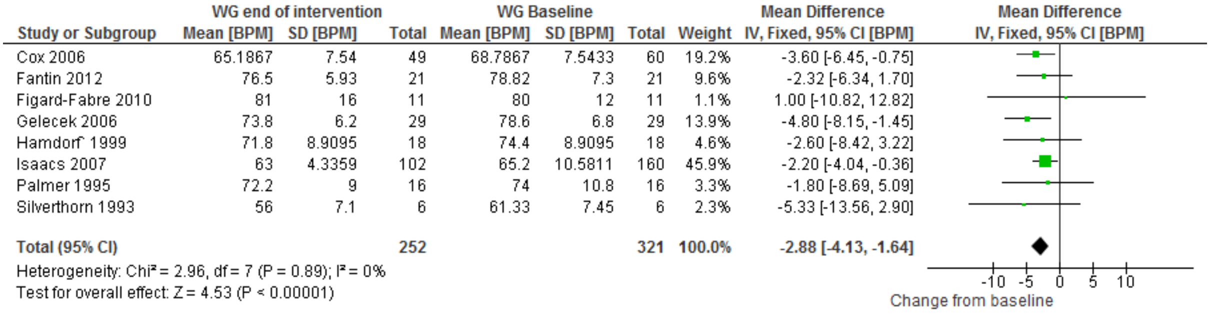

Body fat

Study or Subgroup	WG end of intervention Mean [%]	SD [%]	Total	WG Baseline Mean [%]	SD [%]	Total	Weight	Mean Difference IV, Fixed, 95% CI [%]
Brandon 2006	43.2964	7.0012	28	45.0643	6.9943	28	4.7%	-1.77 [-5.43, 1.90]
Duncan 1991	25.9043	6.7869	46	27.3174	6.6319	46	8.3%	-1.41 [-4.16, 1.33]
Figard-Fabre 2010	39.9	2.4	11	41.1	2.8	11	13.2%	-1.20 [-3.38, 0.98]
Hinkleman1993	36.3	4.2426	18	36.5	4.6669	18	7.4%	-0.20 [-3.11, 2.71]
Isaacs 2007	36.22	2.3139	104	38	8.8176	311	54.0%	-1.78 [-2.86, -0.70]
Moss 2009	26.2545	11.0559	100	25.2	9.4945	100	7.7%	1.05 [-1.80, 3.91]
Song 2013	30.7	5.6	21	31.9	6.2	21	4.9%	-1.20 [-4.77, 2.37]
**Total (95% CI)**			**328**			**535**	**100.0%**	**-1.31 [-2.10, -0.52]**

Heterogeneity: Chi² = 4.00, df = 6 (P = 0.68); I² = 0%
Test for overall effect: Z = 3.25 (P = 0.001)

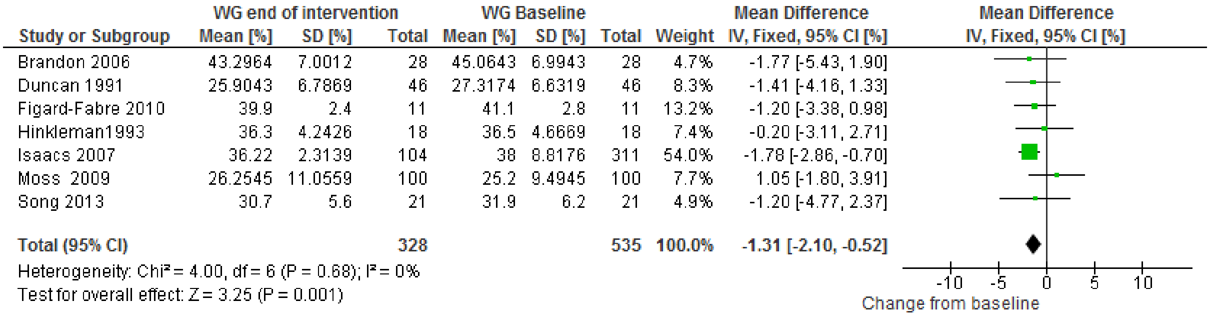

BMI

Study or Subgroup	WG at end of intervention Mean [kg/m2]	SD [kg/m2]	Total	WG Baseline Mean [kg/m2]	SD [kg/m2]	Total	Weight	Mean Difference IV, Fixed, 95% CI [kg/m2]
Brandon 2006	31.4607	7.7792	28	32.125	7.4497	28	1.4%	-0.66 [-4.65, 3.33]
Cox 2006	26.3182	3.5272	49	26.4382	3.5272	60	12.9%	-0.12 [-1.45, 1.21]
Dallachio 2010	21.92	4.9	14	24.07	5.1	14	1.7%	-2.15 [-5.85, 1.55]
Fantin 2012	28.58	3.8	21	28.63	4.69	21	3.4%	-0.05 [-2.63, 2.53]
Figard-Fabre 2010	31.6	2.2	11	32.3	2.6	11	5.7%	-0.70 [-2.71, 1.31]
Fritz 2006	31.2	5.2	17	31.8	5.2	17	1.9%	-0.60 [-4.10, 2.90]
Gusi 2008	29.4	4.2	51	29.7	4.2	51	8.6%	-0.30 [-1.93, 1.33]
Isaacs 2007	29.65	1.0798	104	30.6	6	311	46.9%	-0.95 [-1.65, -0.25]
Moss 2009	26.2545	11.0559	100	29.159	7.608	100	3.3%	-2.90 [-5.53, -0.27]
Negri 2010	28.9	4.2	21	29.2	4.2	21	3.5%	-0.30 [-2.84, 2.24]
Song 2013	23	3.1	21	23.2	2.9	21	6.9%	-0.20 [-2.02, 1.62]
Takahashi 2013	22.1	3.3675	14	22.3	3.3675	14	3.7%	-0.20 [-2.69, 2.29]
**Total (95% CI)**			**451**			**669**	**100.0%**	**-0.71 [-1.19, -0.23]**

Heterogeneity: Chi² = 5.52, df = 11 (P = 0.90); I² = 0%
Test for overall effect: Z = 2.92 (P = 0.003)

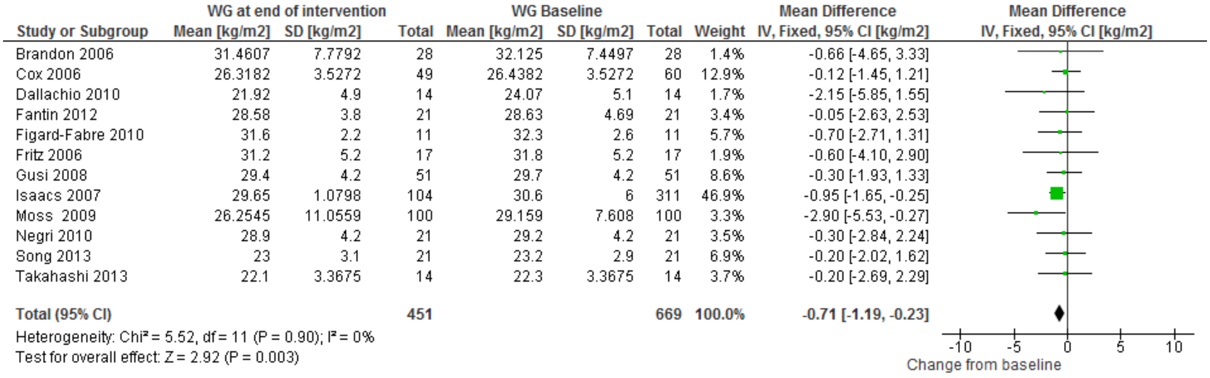

Total cholesterol

Study or Subgroup	WG end of intervention Mean [mmol/L]	SD [mmol/L]	Total	WG Baseline Mean [mmol/L]	SD [mmol/L]	Total	Weight	Mean Difference IV, Fixed, 95% CI [mmol/L]
Duncan 1991	4.7719	0.843	43	4.7902	0.8623	43	8.5%	-0.02 [-0.38, 0.34]
Fantin 2012	5.79	0.91	21	5.87	1.02	21	3.2%	-0.08 [-0.66, 0.50]
Fritz 2006	5.1	1.1	17	5.7	1.1	17	2.0%	-0.60 [-1.34, 0.14]
Gelecek 2006	3.43	0.6	29	3.73	0.46	29	14.7%	-0.30 [-0.58, -0.02]
Hinkleman1993	5.04	1.1455	18	5.07	1.0607	18	2.1%	-0.03 [-0.75, 0.69]
Isaacs 2007	5.68	0.6519	75	5.76	0.07	258	50.8%	-0.08 [-0.23, 0.07]
Morrison 2009	5.1	0.8	19	5.4	0.8	19	4.3%	-0.30 [-0.81, 0.21]
Negri 2010	4.53	0.72	21	4.63	0.83	21	5.0%	-0.10 [-0.57, 0.37]
Park 2013	6.08	0.7249	7	5.15	0.6614	7	2.1%	0.93 [0.20, 1.66]
Song 2013	5	0.6	21	5.2	0.7	21	7.1%	-0.20 [-0.59, 0.19]
**Total (95% CI)**			**271**			**454**	**100.0%**	**-0.11 [-0.22, -0.01]**

Heterogeneity: Chi² = 12.58, df = 9 (P = 0.18); I² = 28%
Test for overall effect: Z = 2.13 (P = 0.03)

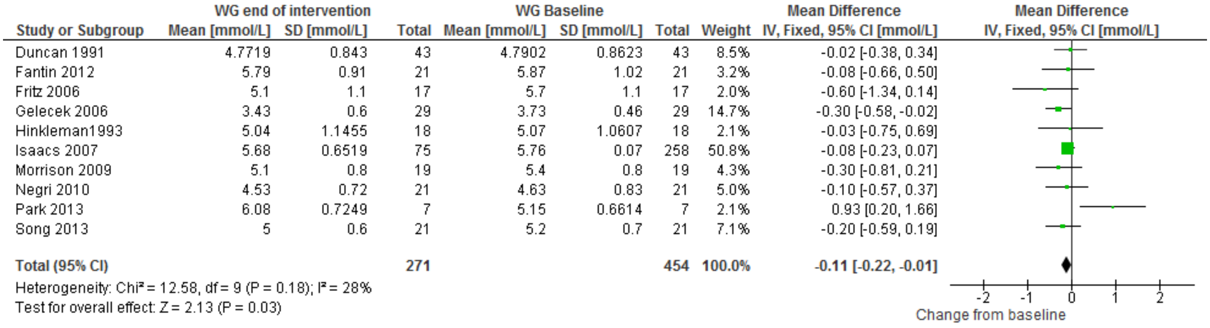

VO2 max.

Study or Subgroup	WG end of intervention Mean [ml/kg/min]	SD [ml/kg/min]	Total	WG Baseline Mean [ml/kg/min]	SD [ml/kg/min]	Total	Weight	Mean Difference IV, Fixed, 95% CI [ml/kg/min]
Armstrong 2004	31.53	5.3	9	25.55	5.5	9	3.9%	5.98 [0.99, 10.97]
Brandon 2006	36.4393	5.3991	28	32.1429	5.5874	28	11.8%	4.30 [1.42, 7.17]
Cox 2006	30.09	4.165	49	27.551	4.165	60	39.5%	2.54 [0.97, 4.11]
Dallachio 2010	33.28	4.7	14	31.14	4.8	14	7.9%	2.14 [-1.38, 5.66]
Duncan 1991	34.5714	5.8685	42	31.6	5.791	42	15.7%	2.97 [0.48, 5.46]
Hinkleman1993	26.3	2.9698	18	25.7	3.8184	18	19.5%	0.60 [-1.63, 2.83]
Silverthorn 1993	45.85	5.95	6	36.16	7.38	6	1.7%	9.69 [2.10, 17.28]
**Total (95% CI)**			**166**			**177**	**100.0%**	**2.66 [1.67, 3.65]**

Heterogeneity: Chi² = 9.67, df = 6 (P = 0.14); I² = 38%
Test for overall effect: Z = 5.28 (P < 0.00001)

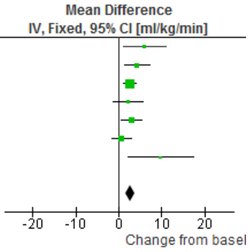

Quality of life SF36 (physical functioning)

Study or Subgroup	WG end of intervention Mean	SD	Total	WG baseline Mean	SD	Total	Weight	Mean Difference IV, Fixed, 95% CI
Brousseau 2012	70.088	18.819	44	63.003	18.332	79	64.3%	7.08 [0.21, 13.96]
Moore-Harrison 2008	85.8	13.6	24	81.7	18.6	24	35.7%	4.10 [-5.12, 13.32]
**Total (95% CI)**			**68**			**103**	**100.0%**	**6.02 [0.51, 11.53]**

Heterogeneity: Chi² = 0.26, df = 1 (P = 0.61); I² = 0%
Test for overall effect: Z = 2.14 (P = 0.03)

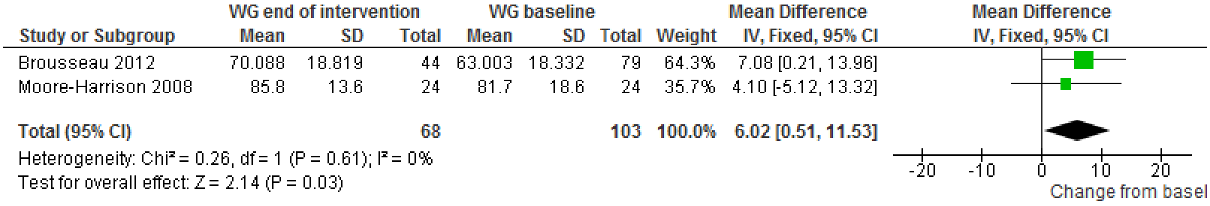

6 minute walk test

Study or Subgroup	WG end of intervention Mean [metres]	SD [metres]	Total	WG Baseline Mean [metres]	SD [metres]	Total	Weight	Mean Difference IV, Fixed, 95% CI [metres]
Brousseau 2012	524.86	106.52	44	456.45	87.62	79	50.5%	68.41 [31.48, 105.34]
Negri 2010	612	78.8	21	521	37.2	21	49.5%	91.00 [53.73, 128.27]
**Total (95% CI)**			**65**			**100**	**100.0%**	**79.60 [53.37, 105.84]**

Heterogeneity: Chi² = 0.71, df = 1 (P = 0.40); I² = 0%
Test for overall effect: Z = 5.95 (P < 0.00001)

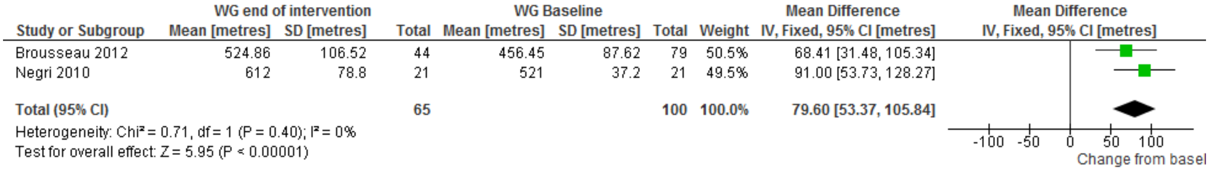

Depression

Study or Subgroup	WG end of intervention Mean	SD	Total	WG baseline Mean	SD	Total	Weight	Std. Mean Difference IV, Fixed, 95% CI	Std. Mean Difference IV, Fixed, 95% CI
Armstrong 2004	6.33	3.67	9	17.25	4	9	4.7%	-2.71 [-4.07, -1.35]	
Dallachio 2010	7.5	3.3	13	13.3	6.7	13	12.7%	-1.06 [-1.89, -0.23]	
Gusi 2008	1.2	0.4	51	1.4	0.6	51	57.0%	-0.39 [-0.78, 0.00]	
Legrand 2009	9.75	2.4138	12	15.335	2.0609	12	7.3%	-2.40 [-3.49, -1.31]	
Palmer 1995	12.2	7.5	16	12.7	10.3	16	18.2%	-0.05 [-0.75, 0.64]	
**Total (95% CI)**			**101**			**101**	**100.0%**	**-0.67 [-0.97, -0.38]**	

Heterogeneity: Chi² = 24.14, df = 4 (P < 0.0001); I² = 83%
Test for overall effect: Z = 4.44 (P < 0.00001)

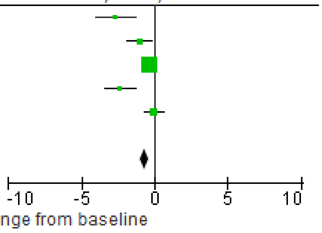

Waist circumference

Study or Subgroup	WG end of intervention Mean [cm]	SD [cm]	Total	WG Baseline Mean [cm]	SD [cm]	Total	Weight	Mean Difference IV, Fixed, 95% CI [cm]	Mean Difference IV, Fixed, 95% CI [cm]
Fantin 2012	87.38	10.28	21	89.33	10.46	21	52.2%	-1.95 [-8.22, 4.32]	
Takahashi 2013	77	9.7283	14	82.3	7.8575	14	47.8%	-5.30 [-11.85, 1.25]	
**Total (95% CI)**			**35**			**35**	**100.0%**	**-3.55 [-8.08, 0.98]**	

Heterogeneity: Chi² = 0.52, df = 1 (P = 0.47); I² = 0%
Test for overall effect: Z = 1.54 (P = 0.12)

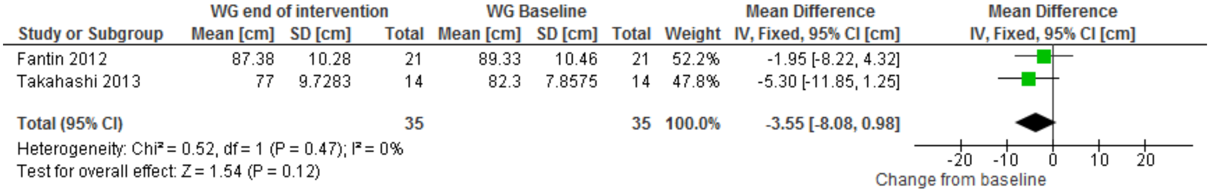

HbA1C

Study or Subgroup	WG end of intervention Mean [%]	SD [%]	Total	WG Baseline Mean [%]	SD [%]	Total	Weight	Mean Difference IV, Fixed, 95% CI [%]	Mean Difference IV, Fixed, 95% CI [%]
Fantin 2012	5.81	0.39	21	5.84	0.38	21	37.2%	-0.03 [-0.26, 0.20]	
Fritz 2006	6.2	0.4463	17	6.3	0.4463	17	22.4%	-0.10 [-0.40, 0.20]	
Negri 2010	7.23	0.64	21	7.5	0.72	21	11.9%	-0.27 [-0.68, 0.14]	
Park 2013	5.21	0.2117	7	5.37	0.291	7	28.4%	-0.16 [-0.43, 0.11]	
**Total (95% CI)**			**66**			**66**	**100.0%**	**-0.11 [-0.25, 0.03]**	

Heterogeneity: Chi² = 1.17, df = 3 (P = 0.76); I² = 0%
Test for overall effect: Z = 1.53 (P = 0.13)

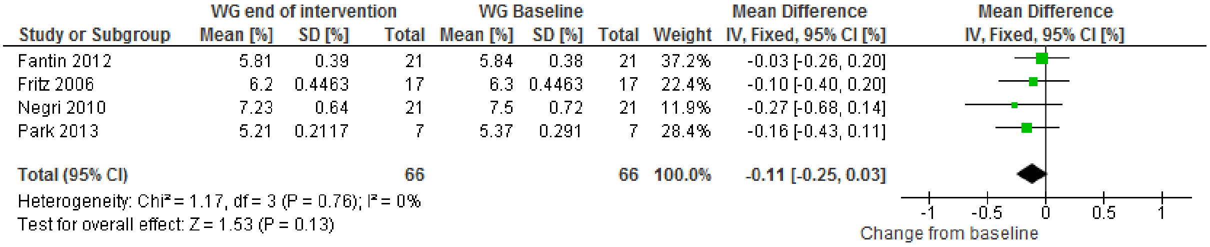

Glucose

Study or Subgroup	WG end of intervention Mean [mmol/l]	SD [mmol/l]	Total	WG Baseline Mean [mmol/l]	SD [mmol/l]	Total	Weight	Mean Difference IV, Fixed, 95% CI [mmol/l]	Mean Difference IV, Fixed, 95% CI [mmol/l]
Fantin 2012	4.9	0.5	21	4.9	0.5	21	40.1%	0.00 [-0.30, 0.30]	
Fritz 2006	9.2	2.8	17	9.2	2.8	17	1.0%	0.00 [-1.88, 1.88]	
Morrison 2009	4.7	0.5	19	4.9	0.4	19	44.3%	-0.20 [-0.49, 0.09]	
Negri 2010	7.8	1.7	21	8.6	2.2	21	2.6%	-0.80 [-1.99, 0.39]	
Park 2013	5.6	0.5292	7	5.4	0.5292	7	11.9%	0.20 [-0.35, 0.75]	
**Total (95% CI)**			**85**			**85**	**100.0%**	**-0.09 [-0.28, 0.11]**	

Heterogeneity: Chi² = 3.33, df = 4 (P = 0.50); I² = 0%
Test for overall effect: Z = 0.87 (P = 0.38)

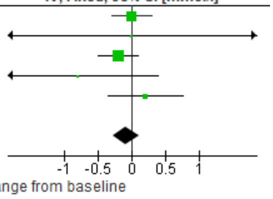

Low density lipids

Study or Subgroup	WG end of intervention Mean [mmol/L]	SD [mmol/L]	Total	WG Baseline Mean [mmol/L]	SD [mmol/L]	Total	Weight	Mean Difference IV, Fixed, 95% CI [mmol/L]	Mean Difference IV, Fixed, 95% CI [mmol/L]
Duncan 1991	2.8386	0.6793	43	2.9314	0.6952	43	13.3%	-0.09 [-0.38, 0.20]	
Fantin 2012	3.74	0.88	21	3.72	0.86	21	4.0%	0.02 [-0.51, 0.55]	
Fritz 2006	2.8	1.2	17	3.4	1.2	17	1.7%	-0.60 [-1.41, 0.21]	
Gelecek 2006	1.87	0.44	29	2.07	0.46	29	20.9%	-0.20 [-0.43, 0.03]	
Hinkleman1993	3.6	1.0607	18	3.51	0.8061	18	3.0%	0.09 [-0.53, 0.71]	
Isaacs 2007	3.46	0.5532	72	3.44	0.9487	250	37.1%	0.02 [-0.15, 0.19]	
Morrison 2009	2.9	0.7	19	3.1	0.6	19	6.5%	-0.20 [-0.61, 0.21]	
Negri 2010	2.61	0.65	21	2.69	0.77	21	6.0%	-0.08 [-0.51, 0.35]	
Park 2013	3.49	0.635	7	2.97	0.4762	7	3.2%	0.52 [-0.07, 1.11]	
Song 2013	3.5	0.9	21	3.4	0.8	21	4.2%	0.10 [-0.42, 0.62]	
**Total (95% CI)**			**268**			**446**	**100.0%**	**-0.05 [-0.16, 0.06]**	

Heterogeneity: Chi² = 8.83, df = 9 (P = 0.45); I² = 0%
Test for overall effect: Z = 0.93 (P = 0.35)

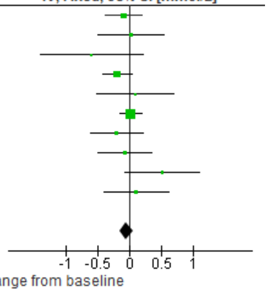

High density lipids

Study or Subgroup	WG end of intervention Mean [mmol/L]	SD [mmol/L]	Total	WG Baseline Mean [mmol/L]	SD [mmol/L]	Total	Weight	Mean Difference IV, Fixed, 95% CI [mmol/L]
Duncan 1991	1.4826	0.3617	43	1.4081	0.3467	43	13.9%	0.07 [-0.08, 0.22]
Fantin 2012	1.42	0.33	21	1.39	0.3	21	8.5%	0.03 [-0.16, 0.22]
Fritz 2006	1.29	0.31	17	1.14	0.31	17	7.2%	0.15 [-0.06, 0.36]
Gelecek 2006	1.19	0.24	29	1.26	0.29	29	16.6%	-0.07 [-0.21, 0.07]
Isaacs 2007	1.35	0.2143	73	1.39	0.48	129	33.5%	-0.04 [-0.14, 0.06]
Morrison 2009	1.7	0.4	19	1.7	0.4	19	4.8%	0.00 [-0.25, 0.25]
Negri 2010	1.34	0.32	21	1.33	0.28	21	9.4%	0.01 [-0.17, 0.19]
Park 2013	1.83	0.4498	7	1.59	0.2646	7	2.1%	0.24 [-0.15, 0.63]
Song 2013	1.7	0.5	21	1.5	0.4	21	4.1%	0.20 [-0.07, 0.47]
**Total (95% CI)**			**251**			**307**	**100.0%**	**0.01 [-0.04, 0.07]**

Heterogeneity: Chi² = 8.04, df = 8 (P = 0.43); I² = 0%
Test for overall effect: Z = 0.45 (P = 0.65)

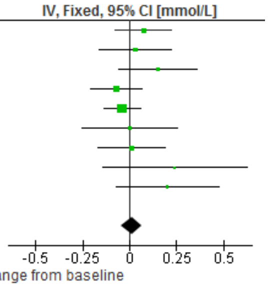

Triglycerides

Study or Subgroup	WG end of intervention Mean [mmol/L]	SD [mmol/L]	Total	WG at Baseline Mean [mmol/L]	SD [mmol/L]	Total	Weight	Mean Difference IV, Fixed, 95% CI [mmol/L]
Duncan 1991	0.997	0.5033	43	1.0014	0.4249	43	12.9%	-0.00 [-0.20, 0.19]
Fantin 2012	1.37	0.69	21	1.64	0.75	21	2.6%	-0.27 [-0.71, 0.17]
Fritz 2006	2.2	1.2	17	2.5	1.2	17	0.8%	-0.30 [-1.11, 0.51]
Gelecek 2006	0.79	0.24	29	0.82	0.16	29	45.5%	-0.03 [-0.13, 0.07]
Hinkleman1993	1.44	0.3818	18	1.11	0.4667	18	6.5%	0.33 [0.05, 0.61]
Isaacs 2007	1.95	0.7823	75	2.09	0.577	75	10.4%	-0.14 [-0.36, 0.08]
Morrison 2009	1	0.2	19	1.3	0.6	19	6.2%	-0.30 [-0.58, -0.02]
Negri 2010	1.23	0.56	21	1.34	0.58	21	4.2%	-0.11 [-0.45, 0.23]
Park 2013	1.27	0.4709	7	1.13	0.3913	7	2.4%	0.14 [-0.31, 0.59]
Song 2013	1.2	0.4	21	1.3	0.4	21	8.6%	-0.10 [-0.34, 0.14]
**Total (95% CI)**			**271**			**271**	**100.0%**	**-0.05 [-0.12, 0.03]**

Heterogeneity: Chi² = 13.39, df = 9 (P = 0.15); I² = 33%
Test for overall effect: Z = 1.25 (P = 0.21)

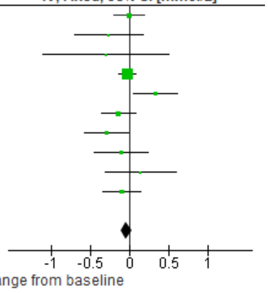

Quality of life SF36 (mental health index)

Study or Subgroup	WG end of intervention Mean	SD	Total	WG at Baseline Mean	SD	Total	Weight	Mean Difference IV, Fixed, 95% CI
Brousseau 2012	78.364	16.007	44	76.5	17.558	78	61.1%	1.86 [-4.26, 7.99]
Moore-Harrison 2008	82	12.9	24	78	14.2	24	38.9%	4.00 [-3.68, 11.68]
**Total (95% CI)**			**68**			**102**	**100.0%**	**2.70 [-2.09, 7.48]**

Heterogeneity: Chi² = 0.18, df = 1 (P = 0.67); I² = 0%
Test for overall effect: Z = 1.10 (P = 0.27)

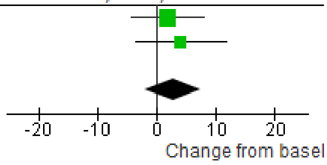

Supplement: Web appendix [file bjsports-2014-094157-s1.pdf]
