# Supplementary figures and images for: Is there evidence that walking groups have health benefits? A systematic review and meta-analysis
Source: Br J Sports Med. 2015 Jan 19;49(11):710–5. doi: 10.1136/bjsports-2014-094157 (PMC4453623; doi:10.1136/bjsports-2014-094157)

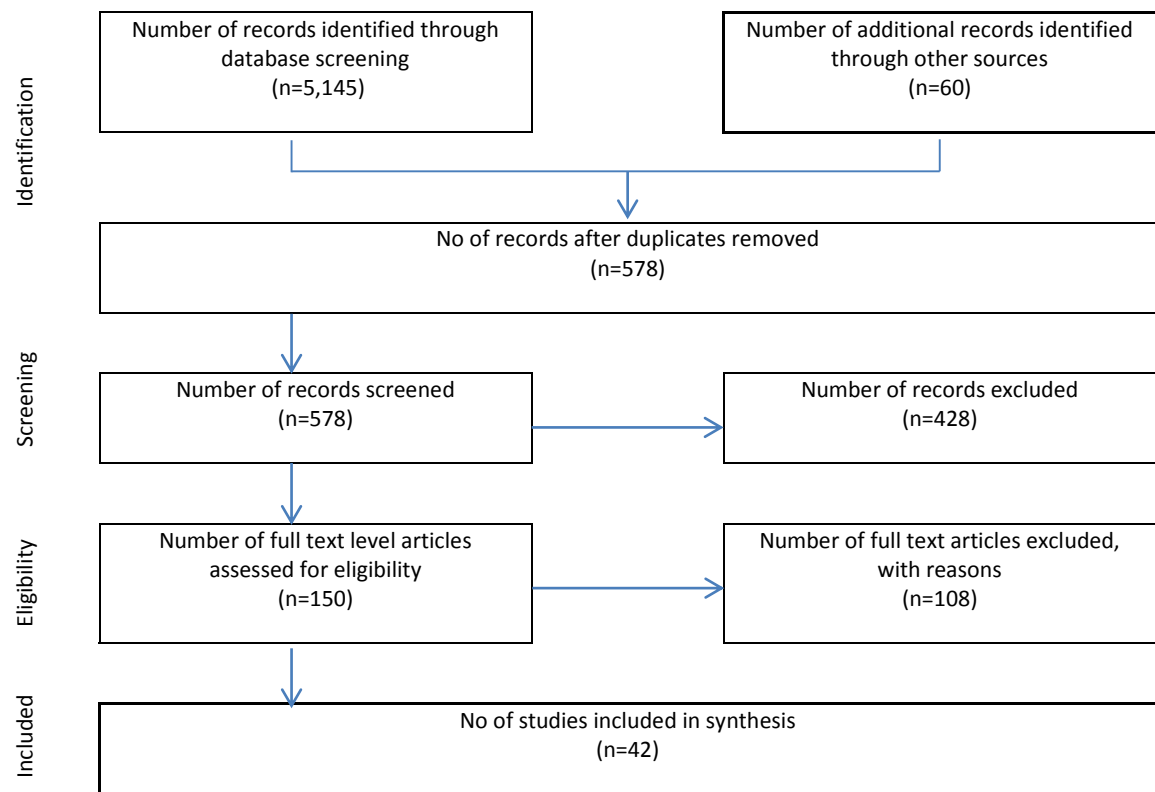

Figure 1: Review flowchart

Supplement: Web figure [file bjsports-2014-094157-s2.pdf]
