# Supplementary material for: Is there evidence that walking groups have health benefits? A systematic review and meta-analysis
Source: Br J Sports Med. 2015 Jan 19;49(11):710–5. doi: 10.1136/bjsports-2014-094157 (PMC4453623; doi:10.1136/bjsports-2014-094157)
Supplement: Web protocol [file bjsports-2014-094157-s3.pdf]

PROSPERO International prospective register of systematic reviews

Review title and timescale

1 Review title

Give the working title of the review. This must be in English. Ideally it should state succinctly the interventions or exposures being reviewed and the associated health or social problem being addressed in the review.

Is there evidence that outdoor walking groups have benefits other than increasing physical activity?

2 Original language title

For reviews in languages other than English, this field should be used to enter the title in the language of the review. This will be displayed together with the English language title.

3 Anticipated or actual start date

Give the date when the systematic review commenced, or is expected to commence.

07/05/2013

4 Anticipated completion date

Give the date by which the review is expected to be completed.

31/01/2014

5 Stage of review at time of this submission

Indicate the stage of progress of the review by ticking the relevant boxes. Reviews that have progressed beyond the point of completing data extraction at the time of initial registration are not eligible for inclusion in PROSPERO. This field should be updated when any amendments are made to a published record.

The review has not yet started ×

Review stage	Started	Completed
Preliminary searches	No	Yes
Piloting of the study selection process	No	Yes
Formal screening of search results against eligibility criteria	No	Yes
Data extraction	Yes	No
Risk of bias (quality) assessment	Yes	No
Data analysis	Yes	No

Provide any other relevant information about the stage of the review here.

This review is part of studentship for a PhD programme.

Review team details

6 Named contact

The named contact acts as the guarantor for the accuracy of the information presented in the register record.

Sarah Hanson

7 Named contact email

Enter the electronic mail address of the named contact.

s.hanson@uea.ac.uk

8 Named contact address

Enter the full postal address for the named contact.

Norwich Medical School Room 1.23 Queens Building University of East Anglia Norwich NR4 7TJ

9 Named contact phone number

Enter the telephone number for the named contact, including international dialing code.

+44 (0)1603 - 593093

10 Organisational affiliation of the review

Full title of the organisational affiliations for this review, and website address if available. This field may be completed as 'None' if the review is not affiliated to any organisation.

Norwich Medical school. University of East Anglia

Website address:

www.uea.ac.uk

11 Review team members and their organisational affiliations

Give the title, first name and last name of all members of the team working directly on the review. Give the organisational affiliations of each member of the review team.

Title	First name	Last name	Affiliation
Mrs	Sarah	Hanson	Norwich Medical School. University of East Anglia
Professor	Andy	Jones	Norwich Medical School. University of East Anglia

12 Funding sources/sponsors

Give details of the individuals, organizations, groups or other legal entities who take responsibility for initiating, managing, sponsoring and/or financing the review. Any unique identification numbers assigned to the review by the individuals or bodies listed should be included.

Not applicable

13 Conflicts of interest

List any conditions that could lead to actual or perceived undue influence on judgements concerning the main topic

investigated in the review.
Are there any actual or potential conflicts of interest?
None known

14 Collaborators

Give the name, affiliation and role of any individuals or organisations who are working on the review but who are not listed as review team members.

Title First name Last name Organisation details

Review methods

15 Review question(s)

State the question(s) to be addressed / review objectives. Please complete a separate box for each question.
Is there evidence that outdoor walking schemes have benefits other than increasing physical activity levels?
What are the characteristics of outdoor walking schemes that show clinical benefits?

16 Searches

Give details of the sources to be searched, and any restrictions (e.g. language or publication period). The full search strategy is not required, but may be supplied as a link or attachment.
A range of health, allied health, physical activity and science databases: AMED EMBASE MEDLINE PsycINFO SportDiscus CINAHL SCOPUS Clinical trials registers Reference lists from included articles will be hand searched Restricted to English language No date restriction Adults only

17 URL to search strategy

If you have one, give the link to your search strategy here. Alternatively you can e-mail this to PROSPERO and we will store and link to it.

I give permission for this file to be made publicly available
Yes

18 Condition or domain being studied

Give a short description of the disease, condition or healthcare domain being studied. This could include health and wellbeing outcomes.
All health and wellbeing outcomes used by the study authors.

19 Participants/population

Give summary criteria for the participants or populations being studied by the review. The preferred format includes details of both inclusion and exclusion criteria.
Inclusion: Adults from the age of 18 Exclusion: Youths and children

20 Intervention(s), exposure(s)

Give full and clear descriptions of the nature of the interventions or the exposures to be reviewed
Inclusion: Interventions where people walk as part of a defined walking intervention Exclusion: Studies that do not involve a walking intervention Inclusion: Where the walking is group based, or where the walking is predominantly group based but participants may also walk on their own to supplement this Exclusion: Participants walking only rarely in groups, or walking on their own e.g. home-based or pedometer based programmes with no group walking Inclusion: Studies that compare group walking with group Nordic walking i.e. group walking can be isolated as an intervention and the outcome directly related to group walking Exclusion: Studies examining Nordic walking only Inclusion: Studies where the outcomes are measures of health status or well-being of participants Exclusion: Studies where the outcomes are solely physical activity e.g. step outcomes / logs of physical activity Inclusion: Studies where the outcome can directly be related to the walking intervention Exclusion: Studies with a mixed intervention (e.g. walking with calcium supplements/walking combined with a health education intervention) where the outcome cannot be isolated and directly attributed to walking

21 Comparator(s)/control

Where relevant, give details of the alternatives against which the main subject/topic of the review will be compared (e.g. another intervention or a non-exposed control group).
There is no comparator.

22 Types of study to be included initially

Give details of the study designs to be included in the review. If there are no restrictions on the types of study design eligible for inclusion, this should be stated.
There is no restriction on study design.

23 Context

Give summary details of the setting and other relevant characteristics which help define the inclusion or exclusion criteria.
Inclusion: Walking outdoors or walking predominantly outdoors but occasionally indoors (e.g. inside tracks or shopping malls for weather reasons). Exclusion: Indoors.

24 Primary outcome(s)

Give the most important outcomes.
All clinical outcomes will be included in the review. This will include physiological outcomes such as blood pressure or lipid profiles. Also included will be psychological, such as quality of life outcomes
Give information on timing and effect measures, as appropriate.
Information will be extracted at the end of the intervention (this may be as little as one month or as long as one year) where this is available.

25 Secondary outcomes

List any additional outcomes that will be addressed. If there are no secondary outcomes enter None.

The characteristics of effective walking groups. This may include whether a walking group, as an intervention, has particularly addressed different socio-economic groups, genders or ethnic minorities.

Give information on timing and effect measures, as appropriate.
This will be a qualitative narrative.

26 Data extraction, (selection and coding)

Give the procedure for selecting studies for the review and extracting data, including the number of researchers involved and how discrepancies will be resolved. List the data to be extracted.
Study selection: All abstracts will be read by the first reviewer and any that do not meet the inclusion will be excluded at this stage. Where adequate information is not provided at abstract level full texts will be evaluated. Where the author has not specified whether the walking group is in fact a walking group or a walking arm of the study, the primary reviewer will contact the author for further information. The second reviewer will review 10% of the papers as a sample to verify that papers have been excluded as per the protocol. Data to be extracted: Author name and date Clinical question addressed Description of the walking group Description of the participants Description of the environment and the provision The number of participants in the study The number of participants in the walking group part of the study The gender of the participants in the walking group Mean age of the walking group Location of the study Description of any socio-economic information Description of ethnicity of the participants The type of walking e.g. self selected, brisk Time in the intervention per week (events x time per week) Dosage of walking group activity in the research (weekly activity x length of time in the study) Results e.g BMI (p 0.257)

27 Risk of bias (quality) assessment

State whether and how risk of bias will be assessed, how the quality of individual studies will be assessed, and whether and how this will influence the planned synthesis.
An eight point tool has been used with 1 point allocated to each element. Randomisation Exposure (no evidence of concurrent intervention) Representativeness Comparability Attrition (over 20% would give a zero score) Follow up tools Precision of the results. This tool will be used by the primary reviewer and the second reviewer will review 10% of the studies. Papers will be presented with their score and also a definition of high quality, medium quality and low quality. No papers will be excluded from the synthesis on quality grounds

28 Strategy for data synthesis

Give the planned general approach to be used, for example whether the data to be used will be aggregate or at the level of individual participants, and whether a quantitative or narrative (descriptive) synthesis is planned. Where appropriate a brief outline of analytic approach should be given.
The results will be given per study on an aggregate level. A table of results will display the extracted information. There will also be a descriptive narrative of the characteristics of walking groups where this information has been available.

29 Analysis of subgroups or subsets

Give any planned exploration of subgroups or subsets within the review. ‘None planned’ is a valid response if no subgroup analyses are planned.
None planned.

Review general information

30 Type of review

Select the type of review from the drop down list.
Intervention

31 Language

Select the language(s) in which the review is being written and will be made available, from the drop down list. Use the control key to select more than one language.
English
Will a summary/abstract be made available in English?
Yes

32 Country

Select the country in which the review is being carried out from the drop down list. For multi-national collaborations select all the countries involved. Use the control key to select more than one country.
England

33 Other registration details

List places where the systematic review title or protocol is registered (such as with he Campbell Collaboration, or The Joanna Briggs Institute). The name of the organisation and any unique identification number assigned to the review by that organization should be included.
None

34 Reference and/or URL for published protocol

Give the citation for the published protocol, if there is one.
Give the link to the published protocol, if there is one. This may be to an external site or to a protocol deposited with CRD in pdf format.

I give permission for this file to be made publicly available
Yes

35 Dissemination plans

Give brief details of plans for communicating essential messages from the review to the appropriate audiences.
Essential messages will be disseminated through journal publication and conference proceedings/presentations

Do you intend to publish the review on completion?
Yes

36 **Keywords**
Give words or phrases that best describe the review. (One word per box, create a new box for each term)
Systematic review
Walking groups
Clinical outcomes

37 **Details of any existing review of the same topic by the same authors**
Give details of earlier versions of the systematic review if an update of an existing review is being registered, including full bibliographic reference if possible.

38 **Current review status**
Review status should be updated when the review is completed and when it is published.
Ongoing

39 **Any additional information**
Provide any further information the review team consider relevant to the registration of the review.

40 **Details of final report/publication(s)**
This field should be left empty until details of the completed review are available.
Give the full citation for the final report or publication of the systematic review.
Give the URL where available.
