# Supplementary material for: Is there evidence that walking groups have health benefits? A systematic review and meta-analysis
Source: Br J Sports Med. 2015 Jan 19;49(11):710–5. doi: 10.1136/bjsports-2014-094157 (PMC4453623; doi:10.1136/bjsports-2014-094157)
Supplement: Web table [file bjsports-2014-094157-s4.pdf]

Table 2: Summary results for all 42 studies

**Lead author**	**Study aim**	**Description of the participants Mean age (SD)**	**Socio-economic (SE) and Ethnicity (E) information Country**	**Description of the environment, provision and group size**	**n = study (walking arm of the study)**	**Type of walking**	**Intervention (as stated or based on average session time)**	**Minutes in the study per person Adherence (where stated)**	**Results. i) Given at the end of the intervention. Difference from baseline. ii) p-values given as stated**
**Armstrong (2004)** [1]	A 12 week RCT to investigate the effects of a pram walking versus a social support group	Had given birth in the past 12 months. Edinburgh postnatal depression scale of ≥ 12<br><br>30	SE: Education and family income information<br>E: not stated<br>Australia	Flat walking path (NB prams) at an area on the Gold Coast.<br>Group size 9. Also encouraged to walk once a week independently	19<br>(9)	Moderate intensity (60-75% of predicted HR).	40 mins.<br>2 times a week for 12 weeks	960<br><br>75%	EPDS↓ (time p <0 .001)<br>VO_2max ↑(time p > 0 .05)
Bjersing (2012) [2]	Effects of 15-week moderate- to high-intensity aerobic exercise (Nordic walking) on the level of serum bioactive IGF-1 in women with fibromyalgia. Low-intensity aerobic exercise (walking) was the control group.	Women with FM aged 20-60 with an interest in exercising outdoors for 15 weeks<br><br>52	Not stated<br>Sweden	Outdoors walking together under the leadership of a physiotherapist.<br>Group size 23	49<br>(23)	Low intensity walking	43 mins.<br>2 times a week for 15 weeks	1290	Pain threshold ↓ (p 0.031)<br>Pain ↓ (p 0.067)<br>6MWT ↑ (p 0.183)<br>IGF-1 ↓ (p 0.148)<br>IGFBP3 ↓ (p 0.881)<br><br>Please see text for sub-group analysis of cerebrospinal markers<br><br>(N.B. walking was the control group)

**Brandon (2006)**<br><br>[3]	Evaluate body composition and blood pressure responses to a 16-week dose of brisk walking in sedentary and obese African American and White women	Sedentary women<br><br>35	SE: not stated<br>E: African American and white<br>USA	Faculty of an urban university and from local government agencies. Outside on courses measured for distance before the study. On rainy days subjects walked on an indoor track or treadmill. Groups of various sizes.	52<br>(28)	16 weeks has been shown to be of sufficient length to provide for significant weight loss. Encouraged to walk briskly at 3.5mph	50 mins.<br>3 times a week for 16 weeks to achieve 3 miles.	2400<br><br><br><br>AA 86%<br>White 90%	African American (AA) and White:<br>Weight:<br>AA ↓(p 0 .543) White ↓(p 0 .001)<br>Body fat:<br>AA↓(p 0 .164) White ↓(p 0 .001)<br>Trunk fat:<br>AA↓(p 0 .024) White ↓(p 0 .001)<br>Leg fat:<br>AA ↓(p 0 .807) White ↓(p 0 .010)<br>BMI:<br>AA↓(p 0 .214) White ↓(p 0 .001)<br>Waist to height ratio<br>AA↓(p 0 .138) White ↓(p 0 .000)<br>SBP:<br>AA ↓(p 0 .001) White↓(p 0 .000)<br>DBP:<br>AA↓(p 0 .001) White↓(p 0 .000)<br>VO_2max :<br>AA↑(p 0 .000)<br>W ↑(p 0 .000)<br><br>(results for AA and white combined in meta-analysis)

**Brosseau (2012)** [4]	Effect of a proven effective walking programme based on the Ottawa Panel clinical practice guidelines implemented through a knowledge translation intervention	Participants with a confirmed diagnosis of mild to moderate unilateral or bilateral osteoarthritis<br><br>63.9<br>(± 10.3)	SE: Level of education given<br>E: White 87.3%, black 1.3%, Hispanic 2.5%, Asian 6.3%, Canada	Two walking sites in Ottawa, Ontario and one in Gatineau, Quebec.<br>71 participants who walked in supervised walking programme but the number in the group not described	222<br>(71)	Ottowa panel evidence based clinical practice guidelines for individuals with osteo-arthritis	55 mins.<br>3 times a week for 52 weeks	8580<br><br>58%	The author gives p-values for walking group versus control. The control group was self-directed using a guidance pamphlet and pedometer and self-recorded<br><br>SF-36:<br>Physical Functioning ↑ (p 0.250)<br>Role physical ↑ (p 0.909)<br>Pain index ↑ (p 0.581)<br>General health perception↓(p 0.223)<br>Vitality ↑ (0.856)<br>Social functioning ↓ (0.266)<br>Role emotional↑ (0.949)<br>Mental Health Index↑ (0.735)<br>Health transition item ↓ (0.821)<br>Standardised physical component ↑(p0.804)<br>Standardized mental component ↑ (p 0.595)<br>AIMS 2:<br>Health perception ↓(0.420)<br>Arthritis impact ↓(0.431)<br>Physical component ↓(0.554)<br>Affect component ↓(0.937)<br>Symptoms component ↓(0.523)<br>Social interaction component ↓(0.081)<br>Role component ↓(0.536)<br>WOMAC :<br>Pain ↓(0.572)<br>Stiffness ↓(0.125)<br>Physical function ↓(0.672)<br>Total WOMAC score ↓(0.612)<br><br>6 minute walk test ↑ (0.063)<br>Gait speed ↓(0.535)<br>Timed up and go ↓(0.770)<br><br>There are also 18 month results given in the paper (all of which have non-significant p values of walking group v control)

Callahan (2011)<br>[5]	Effects of a 6-week walking program for adults with arthritis, Walk With Ease (WWE), delivered in 2 formats, instructor-led group or self-directed	Self-reported joint pain, stiffness, or any type of doctor-diagnosed arthritis. Recruited from urban and rural settings<br><br>70.7 (±9.8)	SE: Education information<br>E: 26% African American 71% white<br>USA - North Carolina	Instructor led group ranged in size from 2 or 3 to 19 participants with most in the range of 5-12 people. Adherence 92.7% versus 83.3% for the self-directed. Participants self-selected the intervention group	462 (192)	Walk with ease (WWE), 6 week community based walking group programme for adults with arthritis	60 mins.<br>3 times a week for 6 weeks	1080<br><br>92.7% versus 83.3% for self - directed	Performance based physical measures:<br>Lower extremity strength, (1 chair and 3 chair stands) in seconds ↓ (improved) ($p < 0.01$)<br>Standing balance/turning ability) in seconds ↓ (improved) ($p < 0.01$)<br>Balance) in seconds ↓ (improved) ($p < 0.01$)<br>Functional mobility:<br>Normal walking speed ↑ ($p < 0.01$), fast walking speed ↑ ($p < .01$)<br>Endurance, 2 minute step test↓ ns<br>Self-reported:<br>HAQ ↓ (improved) ($p < 0.01$)<br>VAS (pain, fatigue, stiffness) ↓ (improved)<br>($p < 0.01$)<br>Pain arthritis self-efficacy ↑ ($p < 0.01$)<br>Symptom arthritis self-efficacy ↑ ($p < 0.05$)<br>Rheumatology attitudes index ↓ (improved) ($p < 0.05$)<br>Self-efficacy for physical activity↑ ns
Cavanaugh (1988)<br>[6]	Evaluate whether brisk walking stops bone loss in post-menopausal women	Recruited via a letter sent to employees at a local university. Post-menopausal 5.6 ± 1.6 years<br><br>55.4 (±1.7)	SE: employment info. given<br>E: not stated<br>USA	Grassy outdoor soccer field. As protocol time increased was also done on city sidewalks. During inclement weather or periods of extreme heat walking was done in building hallways. All of the group (8) met as a group every Monday Wednesday and Friday at noon for 52 weeks	17 (8)	Moderate exercise regime. 60% of target heart rate. Increased time progressively	Average 26 mins.<br>3 times a week for 52 weeks	4056<br><br>73%	Pre exercise heart rate: ↓($p<0.01$)<br>Body fat index ↓<br>Post exercise heart rate: no change<br>Bone loss over 1 year was no different to control.<br><br>Absolute values (and SD) not given within published study therefore unable to include heart rate data in meta-analysis

**Cox (2006)**<br>[7]	Evaluate 6 months of supervised moderate swimming or walking on blood pressure in previously sedentary, normotensive older women.	Women aged 50-70 recruited from media advertising. Sedentary, non-smokers<br><br>55.45 (±4.93)	Not stated<br>Australia	Continuous walk around ovals and parks with a research assistant with a degree in sports science.<br>Usually 4-6 (varied from 2-10)	116<br>(60)	50% of HRreserve and progressed to 60-70% of HRreserve at 8 weeks.	45 mins.<br>3 times a week for 24 weeks	3240<br><br>74.3%	Weight ↓ ns<br>BMI ↓ns<br>Triceps skinfold ↑ns<br>Arm muscle girth ↓ns<br>Urinary sodium excretion ↑ns<br>Urinary calcium excretion ↑ns<br>Systolic BP ↓ns<br>Diastolic BP ↓ns<br>Heart rate ↓($p < 0.001$)<br>VO$_2$max ↑($p < 0.001$)<br><br>Final values not given within published study for BP and therefore unable to include results within meta-analysis<br>(N.B. walking is the control)
Cyarto (2008)<br>[8]	Evaluate and compare resistance training programmes and a group walking programme (control) in improving the functional performance of older adults	Older adults living in retirement villages aged 65-96 years<br><br>78.8 (±6.4)	SE: Level of education stated<br>E: 98% Caucasian<br>Australia	Some hills on the route. Had a leader.<br>Group size 48	167<br>(48)	Walking at a self-selected pace	30 mins<br>2 times a week for 20 weeks	1200<br><br>53%	Chair stand ↑ns<br>Arm curl ↑ns<br>2 minute step test ↑ns<br>Sit and reach↓ ($p < .05$)<br>Back scratch ↑ns<br>Up and go ↓ns<br><br>(N.B. walking is the control)
**Dallocchio (2010)**<br>[9]	A pilot study to evaluate the effects of regular low-medium intensity exercise on sedentary patients with psychogenic movement disorders	Patients with psychogenic movement disorders. Women<br><br>33<br>(±8.79)	Not stated<br>Italy	As a group at a country track. Supervised by the lead investigator. Individually if unable to attend group session.<br>Group size 13	13<br>(13)	Low-moderate intensity walking	Average of 20 mins.<br>3 times a week for 12 weeks	720	PMDRS ↓ (p 0.014)<br>PMDRS function ↓ (p 0.043)<br>BAI ↓ (p 0.034)<br>HDS ↓ (p 0.028)<br>BMI ↓ (p 0.026)<br>VO$_2$max ↑(p 0 .023)<br>Life gratification ↑
**Duncan (1991)**<br>[10]	Whether the quantity and quality of walking necessary to decrease the risk of CVD among women differed substantially from that required to	Women through advertising. Sedentary, randomly selected. 20-40 years of age	SE: Not stated<br>E: 81% white, 17% black and 2% Hispanic<br>USA	Tartan-surfaced 1.6km track.<br>Supervision of an exercise physiologist.<br>Group size 12-18	102<br>(43)	Aerobic walkers (8.0km/hr), Brisk walkers (6.4km/hr) and Strollers (4.8km/hr).	60 mins.<br>5 times a week for 24 weeks	7200<br><br>85% +	BP: Strollers ↓ns / Brisk no change / Aerobic walkers no change<br>Total cholesterol: Strollers ↓ns / Brisk ↓ns / Aerobic walkers ↑ns<br>LDL: Strollers ↓ns / Brisk ↓($p<0.05$) / Aerobic walkers ↑ns<br>HDL: Strollers ↑($p<0.05$)/ Brisk ↑ns/ Aerobic walkers↑ ($p<0.05$)

	improve cardiorespiratory fitness.								Triglycerides: Strollers ↓ns / Brisk No change/ Aerobic walkers↑ ns<br>Cholesterol and HDL ratio: Strollers ↓($p<0.05$) / Brisk ↓ns / Aerobic walkers ↓ns<br>Body fat: all groups ↓ns<br>VO_2max ↑ all groups Strollers (p <0 .05) /Brisk walkers (p <0 .001)<br>Aerobic walkers (p <0 .001)<br><br>(Results combined in the REVMAN programme for meta-analysis)
**Fantin (2012)** [11]	The effect of a moderate (60-min exercise sessions of walking twice per week—approximately 7–8METs per week), 6-month aerobic exercise program on cardiovascular risk factors and pulse wave velocity in a group of apparently healthy elderly women with and without hypertension.	Women living in the community, aged 60-80.<br><br>68.19 (±5.72)	Not stated<br>Italy	Outside and supervised by a qualified physical education instructor.<br>Group size not stated.	21<br>(21)	Brisk walking i.e. moderate physical activity. 7-8 METs/week<br>Increased intensity over time to 75% max heart frequency.	60 mins.<br>2 times a week for 24 weeks	2880	Weight ↑ (p 0.33)<br>BMI ↓ (p 0.81)<br>Waist (circumference) ↓ (p 0.01)<br>SAD ↓ (p 0.04)<br>FM ↓ (p 0.32)<br>FFM ↓ (p 0.33)<br>Glucose ↑ (p 0.30)<br>HbA1C↓ (p 0.15)<br>Total chol ↓ (p 0.64)<br>HDL chol. ↑ (p 0.20)<br>LDL chol ↑ (p 0.92)<br>TG↓ (p 0.02)<br>HR ↓ (p 0.09)<br>SBP ↓ (p 0.31)<br>DBP ↓ (p 0.33)<br>MAP ↓ (p 0.6)<br>PWVcr ↓ (p 0.75)<br>PWVcf ↓ (p 0.02)<br><br>All participants, normotensive and hypertensive.<br>(see text for sub-analysis of normo- and hypertensive)
**Figard-Fabre (2010)** [12]	The effects of a 12 week Nordic interval training programme to those of a walking programme.	Obese middle aged women<br><br>Age not stated	Not stated<br>Italy	Outside and supervised in groups of 12-15 (confirmed by email).	23<br>(11)	Comfortable walking pace and intervals of higher intensity at maximal walking speed	Average 44 minutes.<br>3 times a week for 12 weeks	1584<br><br>81%	Body Mass ↓(p 0.045)<br>BMI ↓(p 0.060)<br>Skinfold thickness ↓(p 0.020)<br>Body fat ↓(p 0.011)<br>HR↑(p 0.048)<br>SBP ↓(p 0.085)<br>DBP↓(p <0.001)

Fisher (2004)<br><br>13	The effects of a neighbourhood walking programme on quality of life of older adults.	Aged over 65, sedentary.<br><br>74.03<br>(± 6.3)	SE: Education and income information<br>E:Black or other. 85% white<br>USA	Leader led walking group in their neighbourhood (28 neighbourhoods for walking). Walking included winter and fall for some groups. Walk leaders recruited locally and paid. Groups of approx. 10 per neighbourhood with 2 walk leaders.	582<br>(280)	Leisurely but purposeful walk	Average 45 mins 3 times a week for 6 months	3510<br><br>74%	SF12: mental and physical scores ↑ ($p < 0.001$)<br>Life satisfaction scores ↑ ($p < 0.001$)<br><br>Absolute values (and SD) not given within published study and therefore unable to include results within QoL meta-analysis
**Fritz (2006)**<br><br>**14**	The effects on metabolic control and cardiovascular risk factors in type 2 diabetes after a period of a low intensity exercise walking programme (walking) feasible to most patients and to the resources of a primary health centre.	Patients with type 2 diabetes from primary care practices suburban communities outside Stockholm.<br><br>60<br>(±7.3)	Not stated<br>Sweden	Walking groups were provided 4 times a week, short distances from the patients' homes. At other times, self-recorded. Typical group size was 10-12. Walks were taken in a rural area, along a "path of health" with no steep elevations. An assistant nurse joined the group during each walk.	52<br>(26)	Low intensity exercise.<br>Brisk walking.<br>To increase their exercise by 45 min of brisk walking, three times weekly, during 4 months.	45 mins.<br>3 times a week for 16 weeks	2160<br><br>65% achieved 80%	Results based on n=17 that achieved 80% of prescribed increased activity<br>SBP ↓($p<0.05$)<br>DBP ↓($p<0.05$)<br>BMI ↓($p<0.05$)<br>HbA1c ↓ ns<br>Fasting glucose no change<br>Fasting insulin ↓ns<br>HOMA2-IR no change ns<br>Total chol ↓($p< 0.05$)<br>HDL cholesterol ↑($p<0.05$)<br>LDL cholesterol ↓($p<0.05$)<br>Triglycerides ↓ns<br>$VO_{2\ max}$ no change (in L/min)<br><br>(See text for analysis of those who did not alter activity levels)
**Gelecek (2006)**<br><br>15	To examine the effects of a 6-week brisk walking training on plasma homocysteine levels and lipid profiles in sedentary young subjects.	Healthy physio-therapy students.<br><br>20 (±2.1)	SE: University students<br>E: Not stated<br>Turkey	Walked in large garden on their campus in 3 groups of: 10, 10 and 9 according to their aerobic capacity determined by sub-maximal cycling test.	29<br>(29)	Brisk walking programme with a speed of 6.4 km/hr	40 mins.<br>3 times a week for 6 weeks	720	Body mass↓($p > 0.05$)<br>SBP↓($p > 0.05$)<br>DBP↓($p > 0.05$)<br>Resting HR↓($p <0 .05$)<br>Homocysteine ↑($p > 0.05$)<br>TG ↓($p > 0.05$)<br>Total –cholesterol↓($p <0 .05$)<br>HDL-c↓($p > 0.05$)<br>LDL-c↓($p <0 .05$)

				Supervised by a physiotherapist.					
**Gusi (2008)**<br><br>[16]	To assess the cost utility of adding a supervised walking programme to the standard "best primary care" for overweight, moderately obese, or moderately depressed elderly women.	Aged 60 and over, moderately depressed or overweight.<br><br>74<br>(±6)	SE: Education and income<br>E:Not stated<br>Spain	Public park or forest tracks with qualified exercise leaders.<br>Socialising encouraged.	107<br>(51)	A pragmatic intervention that could be replicated in a large population	50 mins.<br>3 times a week for 24 weeks	3,600<br><br>86%	BMI ↓ (p 0.003)<br>Geriatric depression scale ↓ (p 0.001)<br>Anxiety (state trait anxiety inventory ↓ (p< 0.001)<br>Anxiety/depression EQ5D ↓ (p 0.009)
**Hamdorf (1999)**<br><br>[17]	The effect of progressive walking programme on healthy women in their 9th decade for evidence of the benefits of exercise.	Recruited through local advertising.<br><br>82.4	Not stated<br>Australia	Outdoors<br>Group size 18.<br>Experienced fitness instructors	38<br>(18)	Low frequency, moderate-intensity, progressive training programme. Target 40-60% of HRR (100bpm)	20 mins.<br>2 times a week for 26 weeks	1040<br><br>89.5%	Resting heart rate ↓(p 0 .029)<br>Exercise heart rate↓(p 0 .002)<br>SBP ↑ ns<br>DBP ↓ns<br>Habitual activity profile and morale: (p values compared to control)<br>MCA and NII↑(improved) (p>0.001)<br>PGMS↑(improved) (p 0.002)
**Hinkleman (1993)**<br><br>[18]	The effects of a walking program on body composition and serum lipids and lipoproteins in overweight women	Recruited from the local community, female aged 24-45 and 10-40% overweight.<br><br>36<br>(±1.6)	Not stated<br>USA	On a measured course near the research testing facility.<br>Supervised.<br>Sessions offered morning and evening.<br>Supervised by an exercise instructor. 2 groups provided for 18 people	36<br>(18)	Brisk walking at 62± 2% VO_2max. 10 second pulse rates or heart monitors used.	45 mins.<br>5 times a week for 15 weeks	3375<br><br>100%	Body fat ↓ns<br>Fat weight ↓ns<br>Lean weight ↑ns<br>Triglycerides ↑ns<br>Cholesterol↓ns<br>LDL –C ↑ns<br>TC/HDL ↓ns
Holmberg (1997)<br><br>[19]	Evaluation of a clinical intervention designed to decrease unsafe wandering and reduce	From a specialised dementia unit with quite significant	Not stated<br>USA	Following the evening meal (6pm) participants walked away from the unit, through public areas of	11<br>(11)	Dementia based rather than physical activity rationale.	90 mins.<br>(including rest stops) Number of times in a week not stated. The intervention	Unable to state from the data given	Measured counts of aggression in a one year period of those who had been involved in the walking group versus no intervention. ↓30%

	interpersonal tension on a dementia unit.	cognitive impairment.<br><br>84.6		the facility (or outside, weather permitting). Walk leaders were lay community volunteers (2 or 3 per group). Groups size average of 10.			lasted for 52 weeks		
**Isaacs (2007)**<br><br>20	The effectiveness and cost-effectiveness of a leisure centre-based exercise programme, a community walking programme and advice on physical activity and local exercise facilities in patients referred for exercise by their GPs	GP referred. 40-74, not physically active and with at least one cardio-vascular risk factor.<br><br>56.9 (±8.5)	SE: Education level, employment status and socio-economic classification given.<br>E: 76% white and 14.3% Asian<br>England	12 different locations (parks and open spaces), 7 days a week with 20 classes to choose from. Started at 9.30 and ran throughout the day until 7.30pm During the winter the evening classes took place under floodlights. Walking classes graded but were free to choose. Trained instructors. 40-50 in each 10 week cohort which facilitated social support and exercise partners.	949 (311) (161 randomised to assess-ment)	60-80% of max. – slightly breathless	60 mins.<br>2 times a week for 10 weeks	1200<br><br>62% attended less than 50%<br>38% attended more than 50%<br>Adherence much higher in those with access to private transport	Changes at 10 weeks: ITT<br>Weight↓ ns<br>BMI ↓ ns<br>% body fat↓ ($p < .001$)<br>Waist-hip ratio no change<br>Resting pulse ↓ ns<br>SBP ↓ ($p < .001$)<br>DBP ↓ (p 0 .06)<br>IKES ↑ ns<br>LEP↑($p <0 .05$)<br>LEP power to weight↑($p <0 .01$)<br>Shoulder abduction↑($p <0 .05$)<br>Cholesterol↓ (p 0.057)<br>HDL↓ ns<br>Cholesterol/HDL↓ ns<br>LDL↓ ns<br>Triglycerides↓ ns<br><br>Please see text for sub-set analysis – 50% randomised to assessment at end of intervention, other time periods and for those on medication.<br><br>Meta-analysis used absolute data from those participants re-randomised (50%) to assessment at 10 weeks. HADS score not included as completed at 6 months rather than end of intervention.

Kamijo (2007)<br><br>[21]	Effects of a 12-week Walking Program on Cognitive Function in Older Adults	Older adults, right handed, sedentary.<br><br>71.1<br>(±1.3)	Not stated<br>Japan	They walked together on the sidewalk that faces the general road with trained exercise personnel.<br>Group size 14.	26<br>(14)	Pace: fairly light to somewhat hard	40 mins.<br>2 times a week for 12 weeks	960<br><br>85%	Reaction time no change<br>Error rate no change<br>Neuro electric measures:<br>P3 amplitude (congruent and incongruent condition):<br>Fz ↑ns<br>C3↑ns<br>Cz↑ns<br>C4↑ns<br>Pz↓ns<br>P3 Latency(congruent and incongruent condition):<br>Fz ↓ns<br>C3↓ns<br>Cz↓ns<br>C4↓ns<br>Pz↓ns
Kayo (2012)<br><br>[22]	To compare the effectiveness of muscle-strengthening exercises and a walking programme in reducing pain and self-reported physical function in patients with fibromyalgia.	Women with fibromyalgia aged between 30-55.<br><br>47.7<br>(±5.3)	SE: schooling<br>E: not stated<br>Brazil	Outdoors or indoors in a gymnasium, depending on the weather.<br>Supervised by a physical therapist.<br>Walking duration and intensity increased over the 16 weeks.<br>Group size not stated but attended the exercise program in small groups, enabling proper supervision.	90<br>(30)	ACSM principles for developing cardio-vascular and muscular fitness and flexibility.	60 mins.<br>3 times a week for 16 weeks	2880	Pain (VAS) ↓ns<br>FIQ ↓($p < 0 .001$) between baseline and week 8. Otherwise ns.<br>SF-36: (NB. Higher score indicates better health outcome)<br>bodily pain score ↑ ($p < 0.01$); general health and vitality ↑ ($p < 0.05$); physical functioning and mental health ↑ ($p < 0.05$)<br>Use of medication: 46.7% restarted medication (80% in the control group)<br><br>SF-36 values not given for end of intervention therefore unable to include in QoL meta-analysis

**Legrand (2009)**<br><br>[23]	The antidepressant effects of two group-based walking programmes (which differed in frequency but not weekly volume) among French older women with subsyndromal depression.	Women, with mild depressive, symptoms, inactive and between 60-74yrs.<br><br>66.8 (±2.5)	Not stated<br>France	Outdoors on a fitness loop of 2/3 of a mile, located in a 1000 acre natural area park. Driven to the site and supervised by the study investigator. 6 in each group.	12<br>(12)	Participants identified their own walk pace (slow, medium, brisk)	60 mins a week (either as one session or 3-5 sessions equating to 60 minutes) for 4 weeks	240<br><br>Above 75%	Geriatric depression scale:<br>Once a week ↓($p < .05$)<br>3-5 times a week ↓($p < .03$)<br><br>(Please see text for qualitative statements and themes from participants)
Mannerkorpi (2010)<br><br>[24]	The effects of moderate-to-high intensity Nordic walking (NW) on functional capacity and pain in fibromyalgia (FM). Low intensity walking is the control	Women aged 20-60 years with fibromyalgia, recruited through advertising.<br><br>50<br>(±7.6)	SE: Education and work status<br>E: Not stated<br>Sweden	Parks and forests with flat areas and small hills under the supervision of a physiotherapist. Group size 33	67<br>(33)	Low-intensity walking ranging from 9 (very light) to 11 (fairly light) on the Borg scale.	20 mins.<br>Once a week for 15 weeks	300<br><br>50%	6 minute walk test ↑ (p 0 .105)<br>Exercise HR ↓ (p 0.079)<br>FIQ pain ↓ (p 0.065)<br>Exercise heart rate ↓ (p 0.079)<br>FIQ physical ↑ (p 0 .929)<br>FIQ total ↑ (p 0 .374)<br>MFI:<br>General fatigue ↓ (p 0.972)<br>Physical fatigue ↓ (p 0.280)<br>Reduced activity ↓ (p 0.194)<br>Reduced motivation ↑ (p 0 .287)<br>Mental fatigue ↓ (p 0.461)<br><br>(NB. walking is control)
McDevitt (2004)<br><br>[25]	To evaluate a 12 week moderate intensity walking programme for sedentary adult outpatients with serious and persistent mental illness.	Adults with serious and persistent mental illness who were enrolled in a psychosocial rehabilitation programme. Volunteers.	SE: not stated<br>E: 60% African American, 27% white, 13% Hispanic<br>USA	Group size 15. No other information.<br><br>41.1 (±12.1)	15<br>(15)	60-79% of HRmax	Average 25 mins.<br>2 or 3 times a week for 12 weeks	750<br><br>76%	SF12 – no change<br>Vigor-activity ↑ (p 0 .05)<br>Mood ↓(improved) (p 0.027)<br>Psychosocial functioning ↑ (p 0 .028)

**Moore-Harrison (2008)**<br><br>26	To describe the population in terms of risk for disability and compare the effects of a walking programme and nutritional education (control) on risk modification and functional performance in lower socioeconomic older adults	26 community dwelling adults aged over 60.<br><br>68.6 (±7.6)	SE: mainly low socio-economic (38% below poverty level). Income (2008) given. Education stated.<br>E: 41.7% African American<br>USA	A cityscape walking path in Athens, Georgia USA. Group size 12	26 (12)	60-75% of HRmax and Borg scale of 12-14	30 mins.<br>3 times a week for 16 weeks	1440<br><br>88.5%	(p value relates to walking v control at 16 weeks)<br>CS – PFP scores:<br>CS-PFP10 total score ↑(p <0 .05)<br>Upper body strength ↑(p <0 .05)<br>Upper body flexibility ↑(p <0 .05)<br>Lower body strength ↑(p <0 .05)<br>Balance & co-ordination ↑(p <0 .05)<br>Endurance ↑(p <0 .05)<br><br>SF-36:<br>Physical Functioning ↑(p 0 .14)<br>Role physical ↑ ns<br>Pain index ↑ ns<br>General health ↑ ns<br>Vitality ↑ ns<br>Social functioning no change ns<br>Role emotional↑ ns<br>Mental Health ↑ ns
**Morrison (2009)**<br><br>27	The effect of an 8-week program of either soft-sand or firm-surface walking on lower limb muscle strength, submaximal fitness, and blood lipid profile in women 60–75 years of age.	Women aged 60-75 and relatively inactive. Randomly assigned.<br><br>65.5 (± 3.7)	Not stated Australia	Participants in the sand-walking group walked on the soft sand at a local beach, well away from the water's edge. The firm-surface-walking group walked on footpaths at the same (beach) locations. Supervised for the 8 weeks by the same person. 19 in each group.	38 (19)	Self- selected speed. Exercise intensity was 74%	Average 33 mins.<br>3 times a week for 8 weeks	792<br><br>83% achieved 64% attendance	Firm surface only.<br>Weight ↑ns<br>SBP ↓ns<br>DBP↓ns<br>Total chol ↓(p < .05)<br>Triglycerides ↓(p < .05)<br>HDL ns<br>LDL ↓ (p < .05)<br>Coronary risk ratio ↓ (p < .05)<br>Glucose ↓ns<br>Strength (kg of force):<br>Knee flexion ↑ns<br>Knee extension ↑ns<br>Knee total ↑ns<br>Hip flexion ↑(p < .05)<br>Hip extension ↑(p < .05)<br>Hip abduction ↑(p < .05)<br>Hip total ↑(p < .05)<br>Total strength ↑(p < .05)<br><br>(Please see text for sand walking results).<br>Results given are for the 38 who attended 64% or more of the sessions.<br>Meta-analysis used firm surface only results

**Moss (2009)**<br><br>[28]	To determine the coronary heart disease (CHD) risk profile of adults with intellectual disabilities residing in a care facility and to determine the effect of a physical activity intervention on the CHD risk profile of the residents.	Men and women with intellectual disabilities residing in a care facility and to determine the effect of a PA intervention on the CHD risk profile of the residents. BMI 29.<br><br>39.2<br>(± 8.9)	Not stated – NB living in a care facility<br>South Africa	400m circular route on the residing grounds with a level walking surface. All 100 walked together with 10 supervisors (post graduate students). 100 walked together	100<br>(100)	Not stated	Average 25 mins.<br>3 times a week for 12 weeks	900<br><br>47%	Body Mass Men↓ns / women ↑ns<br>BMI Men↓ns / women ↓ns<br>WHR Men ↑ns / women ↓ns<br>Body fat ↓ ($p < .05$) (men and women)<br>SBP Men↓ns / women ↓ns<br>DBP Men↓ns / women ↓ns<br>PWC Men ↑($p < .05$) / women ↑ns
**Negri (2010)**<br><br>[29]	The feasibility and effectiveness of an intervention based on the organisation of supervised walking groups	Type II diabetic for 2 years, physically inactive, aged 50-75, A1C 6.5-9.9%<br>Gender not stated.<br><br>65.7<br>(±4.9)	Not stated<br>Italy	A city park supervised by an exercise specialist who encouraged each participant. Walking groups were composed according to walking speed. Max. 20 participants in the group.	60<br>(39)	Low to moderate physical activity intended to achieve an energy expenditure of 10 MET h/week. Groups organised according to walking speed.	45 mins.<br>3 times a week for 16 weeks	2160<br><br>47%	Participants who attended at least 60% of the supervised walking sessions (n= 21):<br>HbA1C ↓ ($p < .05$)<br>Total cholesterol ↓ ($p < .05$)<br>6 min walk time ↑ ($p < .001$)<br>Body weight ↓ns<br>BMI ↓ns<br>HbA1C ↓ ($p < .001$)<br>Total cholesterol ↓ns<br>Glucose ↓ ($p < .05$ compared to control)<br>HDL cholesterol ↑ns<br>LDL cholesterol ↓ns<br>Triglyceride ↓ns<br>SBP ↑ns<br>DBP ↓ns<br><br>Changes to anti-diabetic medication: (compared to control)<br>Dose decreased or discontinued 33% v 5% (p 0.05)<br>Dose increased /No change to regimen ns

Ng (2007)<br>[30]<br>[31]	A pilot study investigating the effectiveness of an adjunctive walking programme in the acute treatment of bipolar disease (2007)<br>___________________<br>Effects of a walking program in the psychiatric in-patient treatment setting: a cohort study (2007)	Private inpatient psychiatric unit.<br><br>45.6 (±16.1)	Not stated Australia	Walks provided on weekday mornings. Even terrain in the vicinity of the hospital which consisted of suburban streets on flat grounds. Group size 6-8.	49 (35)	Not stated	Walks offered for 40 mins.<br>5 times a week.<br>Length of stay in days 19.3 ± 14.	Cannot assess dosage from data given.	Results are for those that reliably attended. Walking is adjunct to treatment. Illness severity at discharge in the walking intervention:<br>CGI-S ↓ ns<br>CGI-I ↓ns<br>Total DASS↓ (p 0 .005)<br>DASS depression↓ (p 0 .048)<br>DASS anxiety↓ (p 0 .002)<br>DASS stress↓ (p 0 .01)<br><br>(retrospective and no data for depression scale meta-analysis)
O'Halloran (2007)<br>[32]	Effects of group walking on mood change in sedentary people with type 2 diabetes.	Sedentary people with type II diabetes.<br><br>54 (±4.7)	Not stated Australia	Three groups available at different locations in metropolitan Melbourne. Group size varied from 6-11.	24 (24)	Moderate level of exertion. Borg scale 10-12	Average 28 mins. Once a week for 6 weeks	168	SEES<br>Positive well-being ↑(p> 0.001)<br>Psychological distress↓(p 0.355)<br>Fatigue ↑ (p 0.061)
O'Hara (2000)<br>[33]	Effects of a walking programme on reducing blood pressure and on increasing health promoting behaviours.	Church based – mid-western African-American. Volunteered. Average BMI 34.2(±5.2)<br><br>41.8 (±7)	SE: Not stated<br>E: African American<br>USA	Group size 14.	14 (14)	Progressive aerobic walking programme (aim 40-75% age adjusted HRmax). Borg scale 12-15	Average 45 mins. 3 times a week for 10 weeks	1350<br><br>80%	SBP ↓<br>DBP ↓<br>(No baseline values or p values given. Insufficient data within published study to include in meta-analysis)
**Palmer (1995)**<br>[34]	Effects of a walking program on attributional style, depression, and self-esteem in women.	Non-exercising, premenopausal female volunteers aged 29-50 recruited	Not stated USA	Met in a university coliseum. Supervised. Group size 16.	27 (16)	60-70% of maximum heart rate (220-age) by carotid pulse	Average of 33 mins.<br>Once a week for 8 weeks	264	SBP↓ns<br>DBP↓ns<br>Pulse↓ns<br>Attributional style:<br>negative events no change<br>positive events ↑ns<br>CES depression ↓ns<br>Rosenberg self-esteem↑ (p<0.05)

		through advertising.<br><br>37.4							VO_2 max↑<br>(unable to include VO_2max into meta-analysis due to limited data)
**Park (2013)**<br><br>[35]	Effects of a low-volume walking programme and vitamin E supplementation on oxidative damage and health-related variables in healthy older adults.	Healthy older adults recruited from the local community.<br><br>71.9<br>(±1.9)	Not stated<br>Japan	Outdoors, supervised by experienced assistants. Walked in the morning. Group size 7.	38<br>(7)	Low volume walking programme of < 150 minutes per week. 48% HR reserve.	44 mins.<br>2 times a week for 12 weeks	1056	Results from control group (i.e. no vitamin E supplementation)<br>Body mass ↑ (p 0.020)<br>BMI ↑(p 0.024)<br>Waist circumference ↑(p 0.603)<br>SBP ↑(p 0.265)<br>DBP ↑(p 0.737)<br>Triacylglycerol ↑ (p 0.109)<br>TC↑ (p 0.001)<br>HDL-C ↑ (p 0.081)<br>LDL-C ↑ (p 0.004)<br>Glucose ↑ (p 0.992)<br>Insulin ↑ (p 0.021)<br>HbA1c ↓ (p 0.001)<br>C-peptide ↑(p 0.001)<br>sE-selectin ↑(p 0.001)<br>sVCAM-1 ↑(p 0.019)<br>plasma TBARS ↓ (p 0.038)<br><br>(This is a sub-set of the Takahashi et al study and therefore only outcomes not included in Takahashi included in meta-analysis)
Reuter (2011)<br><br>[36]	Effects of a flexibility and relaxation programme, walking, and Nordic walking on Parkinson specific disability and health related quality of life.	Mild to moderate Parkinson's disease with no history of falls.<br><br>63<br>(±3.1)	Not stated<br>Germany	One session a week included walking uphill to improve muscle strength. Their partners were also offered 6 training sessions. Group size 30. Supervised by physiotherapists.	90<br>(30)	Not stated	70 mins.<br>3 times a week for 24 weeks	5040<br><br>90%	UPDRS sum score ↓ (improved) (p < .05)<br>UPDRS motor score ↓ (improved) (p < .05)<br>Pain (VAS) ↓(p < .05)<br>PDQ39 ↓ (improved quality of life) (p < .001)

Roberts (1990)<br>[37]	Effects of walking on reaction and movement times among elders	Recruited from seven senior citizen centres.<br><br>71.8<br>(±1.3)	Not stated<br>USA	Indoors during poor weather. Implemented in the fall.<br>Group size 6-10	60<br>(31)	60-70% of age-adjusted maximum HR. increased distance from 0.9 to 1.9 miles	30 mins.<br>3 times a week for 6 weeks	540<br><br>70%	Simple reaction time: ↓ns<br>Choice reaction time: ↓ns<br>Simple movement time: ↓ns<br>Choice movement time: ↓ns
Rooks (1997)<br>[38]	To examine the potential neuromotor benefits of walking in community dwelling older adults	Recruited from a suburban community centre.<br><br>79.2<br>(±4.3)	SE: not stated<br>E: Caucasian<br>USA	Outside in a large parking area, along a wooded path or in a gymnasium depending on the weather. Walked together.<br>Group size 9.	18<br>(11)	Self- paced	Average 37 mins. Three times a week for 16 weeks	1776<br><br>92%	Balance:<br>One-legged stand eyes open ↑(p 0.02)<br>One-legged stand eyes closed↑(p 0.05)<br>Tandem walk↓(p< 0 .01)<br>Mis-steps↓(p 0 .05)<br>Reaction times:<br>Lower extremity↓(p 0 .36)<br>Upper extremity↓(p<0 .96)<br>Knee extension strength: Left ↑(p 0.51).<br>Right ↑(p 0.045)<br>Stair climb ↓(p<0.02)
**<u>Silverthorn (1993)</u>**<br>[39]	Effects of exercise on aerobic capacity and body composition in adults with Prader-Willi syndrome	Adults with Prader-Willi syndrome from two group homes in USA.<br><br>25	Not stated – in residential home<br>USA	A level riverbank trail.<br>Group size 6.	11<br>(6)	Progressively increased pace - 20-23 mins per km, progressed to 13.5-16.5 mins per km	115 mins.<br>2-4 times a week for 24 weeks.	8280	Body weight ↓(p<0.016)<br>Biceps skin fold ↓(p<0.023)<br>Triceps skinfold ↓ns<br>Resting HR ↓(< p 0 .05)<br>VO_2max ↑(< p 0 .05)
**<u>Song (2013)</u>**<br>**<u>[40]</u>**	To compare the effects of Nordic walking programme to those of a normal walking programme on the body composition, muscle strength and lipid profile of women who are over 65 years of age	Women over 65<br><br>68.2<br>(±2.5)	SE: Level of schooling given<br>E: not stated<br>South Korea	A park with a 400 metre track in a metropolitan city. Gym used during inclement weather. Run by person who majored in PE. Intervention ran from February to May.<br>Group size 21	67<br>(21)	Progressed from 11-16 on the Borg scale	60 mins.<br>3 times a week for 12 weeks	2160	Weight↓ (p 0 .002)<br>BMI ↓ (p .257)<br>Total body water ↑ (p 0.626)<br>Skeletal body mass ↑ (p <0.001)<br>Percent body fat ↓ (p 0.005)<br>Grip strength ↑ (< 0.001)<br>Sit to stand (no of times) ↑ (p < 0.001)<br>Arm curls (number of times) ↑ (p < 0.001)<br>Total Cholesterol ↓ (p 0 011)<br>Triglyceride ↓ (p 0 .062)<br>HDL Cholesterol ↑ (p 0.890)<br>LDL Cholesterol ↑ (p 0.860)<br><br>(Walking is the control group)

**Takahashi, (2013)**<br><br>[41]	To examine the effects of a low-volume exercise-training program (100 min/week) on oxidative stress and leukocyte activation marker levels in older adults.	Older adults from the local community. Gender not stated<br><br>67.8<br>(±1.3)	Not stated<br>Japan	In the local community supervised by trainers in the morning (9-10 am) between March and May 2011. The environment was fairly flat road but some parts of road were uphill (but nothing very difficult to walk for older adults). Group size 14	28<br>(14)	Low volume exercise training under the 150 mins. Per week as recommende d by the WHO	50 mins.<br>2 times per week for 12 weeks	1200	Body mass ↓ (p < 0 .01)<br>BMI ↓ (p < 0 .01)<br>Waist circumference ↓ (p < 0 .01)<br>SBP ↓ (p < 0 .01)<br>DBP ↓ (p < 0 .01)<br>AOPP ↓(p 0 .014)<br>SOD ↑ (p 0 .619)<br>CAT↓ (p 0.106)<br>GPX ↑ (p 0.242)<br>TRX ↑ (p 0.444)<br>TNF-α ↑ (p 0.144)<br>IL-1β ↓ (p 0.864)<br>IL-10 ↓ (p 0.094)<br>MPO ↓ (p 0 .101)<br>Calprotectin ↑ (p 0.129)<br>CD66b ↓ (p 0 .001)<br>CD62L ↑ (p < .05)
Thomas (2006)<br><br>[42]	The effect of a Supervised walking programme on wandering among residents with dementia	Nursing home residents selected by the nursing staff with dementia and a 'wanderer'.<br><br>Ranged from 71-89	Not stated<br>USA	The walking environment included other units in the facility, social areas and the outdoor grounds which comprised sidewalks and seated areas surrounding the facility. Reminiscence was used. Late morning walks. A group of 6 and a group of 7.	13<br>(13)	Not stated. Residential in a nursing home	30-40 minutes. Frequency not stated. The study was for 3 weeks	Unable to assess from the data.	General wandering decreased, especially in those in early to middle stages of dementia.
van Uffelen (2007)<br><br>[43]<br><br>[44]<br>[45]	The effects of aerobic exercise or vitamin B supplementation on cognitive function in older adults with mild cognitive impairment (2008)<br>________________	Community-dwelling adults aged 70–80 with mild cognitive impairment recruited via a publicity campaign in a Dutch town.	SE: Level of education stated<br>E: not stated<br>Netherlands	In municipal parks near the subjects' own neighbourhood. Eight classes were started in four districts. 4 trained walking instructors were hired for the	152<br>(77)	Designed to improve aerobic fitness. Moderate intensity (three METs)	60 mins.<br>2 times a week for 52 weeks	6240<br><br>63%	Walking programme v placebo<br><br>MMSE ↓ (men) no change in women ns<br>AVLT 1–5 (words) ↓ (men and women)<br>AVLT 6 (words) ↓ (men and women)<br>SCWT-A task 1 ↑ (men)↓ (women) both<br>SCWT-A task 2 ↓ (men and women)<br>SCWT-A task 3 ↓ (men and women)

	The effects of walking and vitamin B supplementation on quality of life in community dwelling adults with mild cognitive impairment (2007)<br>________________<br>Feasibility and effectiveness of a walking program for community dwelling older adults with mild cognitive impairment (2009)	75 (±2.7)		study. Group size 9-18					DSST (symbols) no change (men) and ↑ (women)<br>VFT (words) ↑ (men and women)<br>Difference between baseline and 12 months<br>D-QoL sumscore no change<br>D-QoL aesthetics ↑ns<br>D-QoL belonging no change<br>D-QoL negative effect↑ns<br>D-QoL positive effect no change<br>D-QoL self esteem ↑ns<br>SF12 – mental component summary↑ns<br>SF12 – physical component summary ↑ns

AIMS2, arthritis impact measurement scale 2,AOPP advanced oxidation protein products, AVLT, auditory verbal learning test (higher score indicates better performance),BAI, Beck anxiety inventory, BMI, body mass index, BPM, beats per minute, CAT, catalase, CES, center of epidemiological studies, CGI-S, Clinical Global impression Severity, CGI-I, Improvement scale, Chol., cholesterol, CS-PFP, continuous scale physical functional performance test, DASS, depression anxiety stress scale, DBP, diastolic blood pressure, D-QoL,dementia quality of life, DSST, digit symbol substitution test, EPDS, Edinburgh postnatal depression scale, F(F)M, fat (free) mass, FIQ, Fibromyalgia impact questionnaire, FM, fat mass, GPX, glutathione peroxidase, HAQ, Patient-Reported Outcomes Measurement Information System Health Assessment Questionnaire, HDL, high-density lipoprotein, HDL-C, high density lipoprotein cholesterol, HDS, Hamilton depression scale, HOMA2-IR: computerized homeostasis model assessment of insulin resistance, HR, heart rate, IGF-1, serum insulin growth factor, IGFBP3, insulin-like binding protein, IKES, isometric knee extensor strength, IL, interleukin, LDL, low-density-lipoprotein, LDL-C, low density lipoprotein cholesterol, LEP, leg extensor power, MCA, maximum current activity, MAP, mean arterial pressure, MFI, Multidimensional Fatigue Inventory, MMSE, mini mental state examination, MPO, myeloperoxidase, NII, Normative impairment index, PDQ39, Parkinson's disease questionnaire 39, PGMS, Philadelphia geriatric morale state, PMDRS, psychogenic movement disorder rating scale, PWC, physical work capacity, PWVcf, pulse wave velocity carotid–femoral, PWVcr, pulse wave velocity carotid–radial, SAD, sagittal abdominal diameter, SBP, systolic blood pressure, SCWT-A, Stroop colour word test–abridged, SEES, 12 item subjective exercise experience scale, sE-selectin, soluble E-selectin, SOD, superoxide dismutase, sVCAM-1, soluble vascular adhesion molecule-1, TBARS,,thiobarbituric acid reactive substances, TC, total cholesterol, TG, triglycerides, TNF-α, tumour necrosis factor alpha, TRX, thioredoxin, UPDRS, unified Parkinson's disease rating scale, VAS, visual analogue scale,VFT, verbal fluency test, WHO, World Health Organisation, WHR, waist to height ratio, WOMAC, Western Ontario MacMaster Osteoarthritis Index

Notes:

1. Results are difference between baseline and the end of the intervention. Results for other time points may be available from the text.
2. There may also be physical activity outcomes within the study and available from the text.
3. Summary table may include additional information provided by the author that cannot be found in the published text.
4. P values given where they were available. Not significant (ns) only used where this is the information that the author has provided.
5. ↑ = increase in a measurement – this may or may not be an improvement.
6. Volume of walking applies to walking with the group as stated in the study. No assumptions have been made about additional walking.
7. **Emboldened and underscored** authors indicates that the study is included in the meta-analysis

w1. Armstrong K and Edwards H. The effectiveness of a pram-walking exercise programme in reducing depressive symptomatology for postnatal women. *International Journal of Nursing Practice*. 2004; 10: 177-94.
w2. Bjersing JL, Dehlin M, Erlandsson M, Bokarewa MI and Mannerkorpi K. Changes in pain and insulin-like growth factor 1 in fibromyalgia during exercise: the involvement of cerebrospinal inflammatory factors and neuropeptides. *Arthritis Research and Therapy*. 2012; 14.
w3. Brandon LJ and Elliott-Lloyd MB. Walking, body composition, and blood pressure dose-response in African American and White women. *Ethnicity and Disease*. 2006; 16: 675-81.
w4. Brosseau L, Wells GA, Kenny GP, et al. The implementation of a community-based aerobic walking program for mild to moderate knee osteoarthritis: a knowledge translation randomized controlled trial: part II: clinical outcomes. *BMC Public Health*. 2012; 12: 1073-.
w5. Callahan LF, Shreffler JH, Altpeter M, et al. Evaluation of group and self-directed formats of the arthritis foundation's Walk With Ease program. *Arthritis Care and Research*. 2011; 63: 1098-107.
w6. Cavanaugh DJ and Cann CE. Brisk walking does not stop bone loss in postmenopausal women. *Bone*. 1988; 9: 201-4.
w7. Cox KL, Burke V, Beilin LJ, Grove JR, Blanksby BA and Puddey IB. Blood pressure rise with swimming versus walking in older women: the Sedentary Women Exercise Adherence Trial 2 (SWEAT 2). *Journal of Hypertension*. 2006; 24: 307-14.
w8. Cyarto EV, Brown WJ, Marshall AL and Trost SG. Comparison of the Effects of a Home-Based and Group-Based Resistance Training Program on Functional Ability in Older Adults. *American Journal of Health Promotion*. 2008; 23: 13-7.
w9. Dallocchio C, Arbasino C, Klersy C and Marchioni E. The effects of physical activity on psychogenic movement disorders. *Movement Disorders*. 2010; 25: 421-5.
w10. Duncan JJ, Gordon NF and Scott CB. Women walking for health and fitness: How much is enough? *Journal of the American Medical Association*. 1991; 266: 3295-9.
w11. Fantin F, Rossi A, Morgante S, et al. Supervised walking groups to increase physical activity in elderly women with and without hypertension: effect on pulse wave velocity. *Hypertension Research*. 2012.
w12. Figard-Fabre H, Fabre N, Leonardi A and Schena F. Efficacy of Nordic Walking in Obesity Management. *International Journal of Sports Medicine*. 2011; 32: 407-14.
w13. Fisher K and Li F. A Community-Based Walking Trial to Improve Neighbourhood Quality of Life in Older Adults: A Multilevel Analysis. *Ann Behav Med*. 2004; 28: 186 - 94.
w14. Fritz T, Wandell P, Aberg H and Engfeldt P. Walking for exercise - Does three times per week influence risk factors in type 2 diabetes? *Diabetes Research and Clinical Practice*. 2006; 71: 21-7.
w15. Gelecek N, Pinar L, Ozdirenc M, et al. Effects of brisk walking program on plasma homocysteine level and lipid profile in sedentary young subjects. / Sedanter genc bireylerde tempolu yurume programinin plazma homosistin duzeyine ve lipid profiline etkileri. *Fizyoterapi Rehabilitasyon*. 2006; 17: 42-6.
w16. Gusi N, Reyes M, Gonzalez-Guerrero J, Herrera E and Garcia J. Cost-utility of a walking programme for moderately depressed, obese, or overweight elderly women in primary care: a randomised controlled trial. *BMC Public Health*. 2008; 8: 231.
w17. Hamdorf PA and Penhall RK. Walking with its training effects on the fitness and activity patterns of 79-91 year old females. *Australian and New Zealand Journal of Medicine*. 1999; 29: 22-8.

w18. Hinkleman LL and Nieman DC. The effects of a walking program on body composition and serum lipids and lipoproteins in overweight women. *Journal of Sports Medicine & Physical Fitness*. 1993; 33: 49-58.
w19. Holmberg SK. Evaluation of a clinical intervention for wanderers on a geriatric nursing unit. *Archives of psychiatric nursing*. 1997; 11: 21-8.
w20. Isaacs AJ, Critchley JA, Tai SS, et al. Exercise Evaluation Randomised Trial (EXERT): a randomised trial comparing GP referral for leisure centre-based exercise, community-based walking and advice only. *Health technology assessment*. 2007; 11: iii.
w21. Kamijo K, Nishihira Y, Sakai T, Higashiura T, Seung-Ryol K and Tanaka K. Effects of a 12-week Walking Program on Cognitive Function in Older Adults. *Advances in Exercise & Sports Physiology*. 2007; 13: 31-9.
w22. Kayo AH, Peccin MS, Sanches CM and Trevisani VFM. Effectiveness of physical activity in reducing pain in patients with fibromyalgia: A blinded randomized clinical trial. *Rheumatology International*. 2012; 32: 2285-92.
w23. Legrand FD and Mille CR. The effects of 60 minutes of supervised weekly walking (in a single vs. 3-5 session format) on depressive symptoms among older women: Findings from a pilot randomized trial. *Mental Health and Physical Activity*. 2009; 2: 71-5.
w24. Mannerkorpi K, Nordeman L, Cider Å and Jonsson G. Does moderate-to-high intensity Nordic walking improve functional capacity and pain in fibromyalgia? A prospective randomized controlled trial. *Arthritis Research and Therapy*. 2010; 12.
w25. McDevitt J, Robinson N and Forest D. A group-based walking program at a psychiatric rehabilitation center. *Psychiatric Services*. 2005; 56: 354-5.
w26. Moore-Harrison TL, Speer EM, Johnson FT and Cress ME. The effects of aerobic training and nutrition education on functional performance in low socioeconomic older adults. *Journal of Geriatric Physical Therapy*. 2008; 31: 18-23.
w27. Morrison K, Braham RA, Dawson B and Guelfi K. Effect of a Sand or Firm-Surface Walking Program on Health, Strength, and Fitness in Women 60-75 Years Old. *Journal of Aging & Physical Activity*. 2009; 17: 196-209.
w28. Moss S. Changes in coronary heart disease risk profile of adults with intellectual disabilities following a physical activity intervention. *Journal of Intellectual Disability Research*. 2009; 53: 735-44.
w29. Negri C, Bacchi E and Morgante S. Supervised walking groups to increase physical activity in type 2 diabetic patients. *Diabetes Care*. 2010; 33: 2333 - 5.
w30. Ng F, Dodd S and Berk M. The effects of physical activity in the acute treatment of bipolar disorder: A pilot study. *Journal of Affective Disorders*. 2007; 101: 259-62.
w31. Ng F, Dodd S, Jacka FN, Leslie E and Berk M. Effects of a walking program in the psychiatric in-patient treatment setting: a cohort study. *Health Promotion Journal of Australia*. 2007; 18: 39-42.
w32. O'Halloran P. Mood Changes in Weeks 2 and 6 of a Graduated Group Walking Program in Previously Sedentary People with Type 2 Diabetes. *Aust J Prim Health*. 2007; 13: 68 - 73.
w33. O'Hara RB, Baer JT, Pohlman RL and Laubach LL. The effect of a walking program on blood pressure response in African-American women. *ACSM's Health & Fitness Journal*. 2000; 4: 20-4.
w34. Palmer LK. Effects of a walking program on attributional style, depression, and self-esteem in women. *Perceptual & Motor Skills*. 1995; 81: 891-8.
w35. Park J-H, Miyashita M, Takahashi M, et al. Effects of low-volume walking programme and vitamin E supplementation on oxidative damage and health-related variables in healthy older adults. *Nutrition & metabolism*. 2013; 10: 38.

w36. Reuter I, Mehnert S, Leone P, Kaps M, Oechsner M and Engelhardt M. Effects of a flexibility and relaxation programme, walking, and nordic walking on Parkinson's disease. *Journal of Aging Research*. 2011; 2011: 232473.
w37. Roberts BL. Effects of walking on reaction and movement times among elders. / Effets de la marche sur les temps de reactions et de mouvement chez des personnes agees. *Perceptual & Motor Skills*. 1990; 71: 131-40.
w38. Rooks DS, Ransil DJ and Hayes WC. Self-paced exercise and neuromotor performance in community-dwelling older adults. / Exercice personnalise et performance neuromotrice chez des adultes vivant dans des habitations collectives. *Journal of Aging & Physical Activity*. 1997; 5: 135-49.
w39. Siverthorn KH and Hornak JE. Beneficial effects of exercise on aerobic capacity and body composition in adults with Prader-Willi syndrome. *American Journal on Mental Retardation*. 1993; 97: 654-8.
w40. Song MS, Yoo YK, Choi CH and Kim NC. Effects of nordic walking on body composition, muscle strength, and lipid profile in elderly women. *Asian Nursing Research*. 2013; 7: 1-7.
w41. Takahashi M, Miyashita M, Kawanishi N, et al. Low-volume exercise training attenuates oxidative stress and neutrophils activation in older adults. *European Journal of Applied Physiology*. 2013; 113: 1117-26.
w42. Thomas DW, Glogoski C and Johnson J. The Effect of a Supervised Walking Program on Wandering Among Residents with Dementia. *Activities, Adaptation & Aging*. 2006; 30: 1-13.
w43. van Uffelen JGZ, Chinapaw MJM, van Mechelen W and Hopman-Rock M. Walking or vitamin B for cognition in older adults with mild cognitive impairment? A randomised controlled trial. *British Journal of Sports Medicine*. 2008; 42: 344-51.
w44. Van Uffelen JGZ, Chin APMJM, Hopman-Rock M and Van Mechelen W. The effect of walking and vitamin B supplementation on quality of life in community-dwelling adults with mild cognitive impairment: A randomized, controlled trial. *Quality of Life Research*. 2007; 16: 1137-46.
w45. van Uffelen JGZ, Chinapaw MJM, Hopman-Rock M and van Mechelen W. Feasibility and effectiveness of a walking program for community-dwelling older adults with mild cognitive impairment. *Journal of Aging & Physical Activity*. 2009; 17: 398-15.
